# Boosting Natural Killer Cell-Mediated Targeting of Sarcoma Through DNAM-1 and NKG2D

**DOI:** 10.3389/fimmu.2020.00040

**Published:** 2020-01-28

**Authors:** Ece Canan Sayitoglu, Anna-Maria Georgoudaki, Michael Chrobok, Didem Ozkazanc, Benjamin J. Josey, Muhammad Arif, Kim Kusser, Michelle Hartman, Tamara M. Chinn, Renee Potens, Cevriye Pamukcu, Robin Krueger, Cheng Zhang, Adil Mardinoglu, Evren Alici, Harry Thomas Temple, Tolga Sutlu, Adil Doganay Duru

**Affiliations:** ^1^Dr. Kiran C. Patel College of Allopathic Medicine, Nova Southeastern University, Fort Lauderdale, FL, United States; ^2^NSU Cell Therapy Institute, Nova Southeastern University, Fort Lauderdale, FL, United States; ^3^Center for Hematology and Regenerative Medicine, Karolinska University Hospital Huddinge, Karolinska Institutet, Stockholm, Sweden; ^4^Faculty of Engineering and Natural Sciences, Sabanci University, Istanbul, Turkey; ^5^Science for Life Laboratory, KTH Royal Institute of Technology, Stockholm, Sweden; ^6^Translational Research and Economic Development, Nova Southeastern University, Fort Lauderdale, FL, United States; ^7^Dr. Kiran C. Patel College of Osteopathic Medicine, Nova Southeastern University, Fort Lauderdale, FL, United States; ^8^Faculty of Dentistry, Oral & Craniofacial Sciences, Centre for Host-Microbiome Interactions, King's College London, London, United Kingdom; ^9^Department of Surgery, Dr. Kiran C. Patel College of Allopathic Medicine, Nova Southeastern University, Fort Lauderdale, FL, United States; ^10^Department of Molecular Biology and Genetics, Bogaziçi University, Istanbul, Turkey; ^11^Science for Life Laboratory, Department of Medicine Solna, Karolinska Institutet, Stockholm, Sweden

**Keywords:** cancer immunotherapy, cancer immunology, sarcoma, natural killer (NK) cell, DNAM-1 (CD226), NKG2D (Natural killer group 2 member D), NK-92 cell line

## Abstract

Sarcomas are malignancies of mesenchymal origin that occur in bone and soft tissues. Many are chemo- and radiotherapy resistant, thus conventional treatments fail to increase overall survival. Natural Killer (NK) cells exert anti-tumor activity upon detection of a complex array of tumor ligands, but this has not been thoroughly explored in the context of sarcoma immunotherapy. In this study, we investigated the NK cell receptor/ligand immune profile of primary human sarcoma explants. Analysis of tumors from 32 sarcoma patients identified the proliferative marker PCNA and DNAM-1 ligands CD112 and/or CD155 as commonly expressed antigens that could be efficiently targeted by genetically modified (GM) NK cells. Despite the strong expression of CD112 and CD155 on sarcoma cells, characterization of freshly dissociated sarcomas revealed a general decrease in tumor-infiltrating NK cells compared to the periphery, suggesting a defect in the endogenous NK cell response. We also applied a functional screening approach to identify relevant NK cell receptor/ligand interactions that induce efficient anti-tumor responses using a panel NK-92 cell lines GM to over-express 12 different activating receptors. Using GM NK-92 cells against primary sarcoma explants (*n* = 12) revealed that DNAM-1 over-expression on NK-92 cells led to efficient degranulation against all tested explants (*n* = 12). Additionally, NKG2D over-expression showed enhanced responses against 10 out of 12 explants. These results show that DNAM-1^+^ or NKG2D^+^ GM NK-92 cells may be an efficient approach in targeting sarcomas. The degranulation capacity of GM NK-92 cell lines was also tested against various established tumor cell lines, including neuroblastoma, Schwannoma, melanoma, myeloma, leukemia, prostate, pancreatic, colon, and lung cancer. Enhanced degranulation of DNAM-1^+^ or NKG2D^+^ GM NK-92 cells was observed against the majority of tumor cell lines tested. In conclusion, DNAM-1 or NKG2D over-expression elicited a dynamic increase in NK cell degranulation against all sarcoma explants and cancer cell lines tested, including those that failed to induce a notable response in WT NK-92 cells. These results support the broad therapeutic potential of DNAM-1^+^ or NKG2D^+^ GM NK-92 cells and GM human NK cells for the treatment of sarcomas and other malignancies.

## Introduction

Sarcomas are a group of rare, heterogeneous, and aggressive tumors of mesenchymal origin that may arise in a range of different tissues, including bone, cartilage, connective tissue, muscle, fat, vasculature, and peripheral nerves. While the standard treatment options of chemotherapy, radiotherapy, and surgery efficiently control localized tumors, ~40% of cases experience tumor relapse and metastatic spread with current treatments remaining ineffective in increasing overall survival (1–4). The heterogeneity of sarcoma subtypes and the fact that most therapies currently prove suboptimal, underline the importance of investigations aiming to develop novel targeted treatment approaches.

Both experimental and clinical data support the involvement of the immune system in sarcoma tumorigenesis as immunosuppressed individuals present with a higher risk (5). Moreover, there is evidence for both spontaneous regression as well-efficient immunosurveillance in sarcoma, prompting investigators to explore immunotherapy as a treatment modality (6, 7). Recently, several clinical trials have included sarcoma patients in the testing of various immunotherapy strategies, including: (i) immune checkpoint blockade (8–10); (ii) tumor-specific or tumor-associated peptide vaccines (11–14); and (iii) adoptive immune cell therapies with allogeneic NK cells (ongoing clinical trials), autologous T cells (15), CAR-T cells (ongoing clinical trials) and NY-ESO-1-specific T cells (16) to name a few (17). However, these studies aim to target general tumor markers found in a variety of solid tumors and hematological malignancies, not specifically targeting sarcomas. This highlights the need for more precise tumor characterization, targeted immunomodulation of the individual tumor microenvironment and targeting of sarcoma-specific molecules.

NK cells are promising candidates for cancer immunotherapy, as engagement of their activating receptors with ligands expressed on targets leads to rapid response and efficient elimination of tumor cells (18). In general, NK cell infusions have proven to be well-tolerated and safe with minimal side effects (19). However, modulation of ligand expression in order to avoid NK cell detection is a well-known strategy employed by tumors to escape immune recognition (20). Known ligands for NK cell receptors are expressed on several tumors, including some subtypes of sarcomas. More specifically, proliferating cell nuclear antigen (PCNA) has been shown to be an inhibitory ligand of natural cytotoxicity receptor NKp44 (21, 22). It is also associated with poor prognosis in an analysis covering 16 studies with a total of 691 osteosarcoma patients (23). Osteosarcomas have been previously shown to express CD155 (Poliovirus Receptor, PVR) (24), which is one of the ligands for DNAX accessory molecule-1 (DNAM-1 or CD226), an activating receptor expressed on NK cells, monocytes and a subset of T cells. Rhabdomyosarcoma (RMS) cell lines have been shown to express CD112 (Nectin-2) along with CD155, both ligands of DNAM-1 (25, 26). They also expressed KIR-ligand HLA-I but had low expression of NKG2D ligands MICA/MICB (27). However, these studies focused on a limited group of sarcoma subtypes and NK cell receptors, thus, better characterization of the individual sarcoma tumor's expression of various NK cell ligands could identify relevant receptor/ligand interactions that could mediate efficient NK cell cytotoxic activity.

In this study, we demonstrate an inherent immune defect in the tumor infiltrating NK cell compartment of sarcoma tumors. This prompted the investigation of NK cell ligand expression on fresh and *in vitro* propagated primary sarcoma explants, which identified the presence of DNAM-1 ligands CD112 and CD155. We developed a novel cell-based screening platform which allowed the identification of tumor-specific NK cell receptor engagements. This platform, together with extensive flow cytometry-based characterization of rapidly processed fresh sarcoma surgical material and respective short-term cultured primary human sarcoma explants, were used to identify targetable NK cell receptor/ligand interactions in sarcoma.

Our results show that over-expression of the activating receptor DNAM-1 or NKG2D on NK-92 cells induces efficient anti-sarcoma responses *in vitro* by amplifying the interaction with prevalent ligands CD112 and CD155 or MICA/B and ULBP1-5, respectively, on sarcoma and other tumor cells. This way of arming NK cells against tumor targets that they would otherwise remain inert against, provides a promising novel cellular immunotherapy strategy that can easily be translated to the clinic and has the potential to significantly improve sarcoma treatment.

## Materials and Methods

### Patient Material

Primary sarcoma tumors and blood were collected at the Center for Orthopedic Innovations of the Mercy Miami Hospital, Florida according to rules and regulation specified under Nova Southeastern University Institutional Review Board (protocol # 2017-304).

### Primary Sarcoma Explant Generation From Patient Material

Sarcoma tumor samples were processed within 12 h of surgical excision with the Miltenyi Tumor Dissociation Kit to obtain homogenous cell suspensions in RPMI medium (Gibco) using the Miltenyi GentleMACS Octo Dissociator with heaters. Homogenous cell suspensions were seeded in complete DMEM medium [DMEM (high glucose, GlutaMAX, Gibco) 10% FBS (Gemini Bio-Products), supplemented with 1X non-essential amino acids (NEAA), 1X Antibiotic-Antimycotic and 25 mM HEPES (all from Gibco)] which was changed every 4 days during the first 2 weeks. After 2 weeks in culture, serial passaging is performed based on confluency for the selection of adherent cells. Multiple passages were vitally frozen along the process of explant generation, which was considered complete at passage 12.

### Cell Culture

Primary sarcoma explants were cultured in complete DMEM as explained above. Culture media was renewed once a week, splits were done based on confluency, predictably every 7–10 days. All cell lines except for 293FT (Thermo Scientific), A375 and DM6 were obtained from ATCC. DM6 cells were a kind gift from Dr. Hilliard F. Seigler (Department of Immunology, Duke University Medical Center) and A375 cells were a kind gift from Prof. Michael Nishimura (Loyola University Chicago). U-2 OS and Saos-2 cell lines used during the revision of the manuscript were kind gifts provided by Uygar Tazebay (Gebze Technical University) and Mehmet Öztürk (Izmir Biomedicine and Genome Center). NK-92 cells (ATCC CRL-2407) were cultured in CellGro SCGM (Cellgenix) supplied with 20% FBS and 1,000 U/ml IL-2 (Miltenyi Biotech). K562, THP-1, LNCaP, PC-3, ARH-77, RPMI8226, DM6, and A375 cells were cultured in RPMI (GlutaMAX, Gibco) supplied with 10% FBS. U266 cells were cultured in RPMI (GlutaMAX, Gibco), supplied with 15% FBS. 293FT (Thermo Fisher Scientific), MeWo, SK-MEL-28, sNF02.2, Capan-2 cells were cultured in DMEM (GlutaMAX, Gibco) supplied with 10% FBS. Saos-2 and U-2 OS cells were cultured in McCoy's 5A Medium (ATCC) supplied with 15% and 10% FBS, respectively. SH-SY5Y cells were cultured in EMEM (ATCC) supplied with 10% FBS. Caco-2 cells were cultured in EMEM (ATCC) supplied with 20% FBS. A549 cells were cultured in F-12K medium (ATCC) supplied with 10% FBS.

### Flow Cytometry-Based Phenotyping of Sarcoma Primary Explants and Other Cell Lines

Single cell suspensions of the generated primary sarcoma explants were dissociated using 0.05% trypsin at 37°C, and manual scraping when necessary. The cells were stained with Live/Dead fixable Aqua in PBS for dead cell discrimination (Invitrogen). Surface stainings were performed in BD Horizon Brilliant Stain Buffer (BD Biosciences) according to the manufacturer's instructions. Stained cells were washed in FACS buffer (PBS, 2% FBS, 2 mM EDTA) twice before assessed. Data were acquired on a BD LSR Fortessa X-20 flow cytometer and analyzed using the FlowJo software v10.1 (BD Biosciences). The antibodies from Biolegend and BD Biosciences that were used for stainings are listed in Table S2.

### Intracellular Cytokine Staining

The ability of WT, DNAM-1, and NKG2D modified NK-92 cells to produce IFNγ and TNFα upon 4 h of co-culture of 200,000 NK-92 cells with 200,000 primary sarcoma explant lines or the cell line Saos-2, was assessed by intracellular cytokine staining using the BD cytofix/cytoperm kit according to the manufacturer's instructions. The following antibodies from Biolegend were used at the recommended amounts: IFNγ PE (B27), TNFα APC (MAb11), PE mouse IgG1, κ isotype control (MOPC-21) in PE and APC. BD biosciences: CD56 BV421 (NCAM16.2). For data acquisition the BD LSR Fortessa X-20 flow cytometer were used. Data were analyzed with the FlowJo software v10.1 (BD Biosciences).

### Ligand Expression Characterization Using NKG2D-Fc Chimeric Protein

In order to assess the presence of NKG2D ligands on the surface of primary sarcoma explant lines, we used an NKG2D-Fc chimeric protein where the receptor was fused to the Fc of a human IgG1 (1299-NK, R&D Systems). NKG2D-Fc was reconstituted at 100 μg/ml in PBS. 2 μg/ml of NKG2D-Fc were pre-complexed with 4 μg/ml of mouse-anti-human IgG secondary antibody PE (clone HP6017, Biolegend) in FACS buffer for 1 h on ice. The cells were prepared at 2 × 10^5^ cells per well in a 96-well plate, washed and resuspended in 200 μl of the precomplexed mix (or just secondary antibody as control) and incubated on ice for 30 min. The samples were washed twice with FACS buffer and acquired on the BD LSR Fortessa X-20 flow cytometer. Data were analyzed using FlowJo software v10.1 (BD Biosciences) and normalized to unstained control.

### qPCR-Based Expression Analysis

Primers were selected from the Harvard Medical School PrimerBank (https://pga.mgh.harvard.edu/primerbank/) using the Entrez gene numbers obtained from the Human Protein Atlas. Entrez No's: *ULBP1* #80329, *ULBP2* #80328, *ULBP3* #79465 (https://www.proteinatlas.org/). Primer IDs: *ULBP1* #56181385c1, *ULBP2* #13376824a3, *ULBP3* #13375655c1. Exon-spanning primers capable of detecting multiple transcript variants were selected when possible. Primers were synthesized by Midlands Certified Reagents (Midland, TX).

#### ULBP1

Forward: TAAGTCCAGACCTGAACCACA

Reverse: TCCACCACGTCTCTTAGTGTT

#### ULBP2

Forward: GTGGTGGACATACTTACAGAGC

Reverse: CTGCCCATCGAAACTGAACTG

#### ULBP3

Forward: TCTATGGGTCACCTAGAAGAGC

Reverse: TCCACTGGGTGTGAAATCCTC

RNA was collected and purified with the E.Z.N.A. HP Total RNA Kit (OmegaBioTek R6812-02) and eluted to 40 μl. The cDNA was synthesized with qScript cDNA SuperMix (Quantabio 95048-025). Briefly, 100 ng of RNA was combined with 8 μl qScript cDNA SuperMix and water added to 20 μl total reaction volume. The reaction was thermal-cycled at 25°C for 5 min, 42°C for 30 min, 85°C for 5 min and held at 4°C. Using the PerfeCTa SYBR Green FastMix Low ROX (QuantaBio 95074-250), RT-PCR reactions were carried out in triplicate on 96-well plates (VWR 82006-664) using an AriaMX Real-Time PCR system (Agilent). Each reaction contained: 10 μl of SYBR Green master mix, 5 μl of 100 ng/μl template cDNA, 10 μM of primers, then water added for a 20 μl total reaction volume. Reactions were heated to 95°C for 3 min, 40 cycles of 95°C for 15 s, 60°C for 1 min, and 76°C for 15 s. Following cycling, the reactions were held at 4°C until use. The melting curve at 95°C for 30 s, 65°C for 30 s, and 95°C for 30 s showed no primer dimers. Housekeeping: *GAPDH* in triplicate. Calibrators: *ULBP1- THP1, ULBP2* & *3- U2OS, ULBP4* & *5- Capan-2*.

### The Cancer Genome Atlas Database (TCGA) Analysis

We retrieved TPM table of sarcoma data from TCGA (28) to validate the gene expression of the ligands of NK cell receptors DNAM-1 and NKG2D. Based on the TPM value of each gene, we classified the patients into two groups and examined their prognoses. The prognosis of each group of patients was examined by Kaplan-Meier survival estimators, and the survival outcomes of the two groups were compared by log-rank tests. To choose the best TPM cut-offs for grouping the patients most significantly, all TPM values from the 20th to 80th percentiles were used to group the patients, significant differences in the survival outcomes of the groups were examined and the value yielding the lowest log-rank P-value is selected (28).

### Generation of Genetically Modified NK-92 Cells

For all NK-92 receptor transgenes, codon-optimized cDNA was cloned upstream of IRES in LeGO-iG2 vector under the control of the SFFV promoter (LeGO-iG2 was a kind gift from Boris Fehse, Addgene #27341) (29). Lentiviral vector production and transduction were performed as before (30). Briefly, NK-92 cells were seeded at 5 × 10^5^ cells/ml in viral supernatant containing the gene-of-interest-iG2 virus for 6 h in the presence of 6 μM BX795 (Sigma-Aldrich). Next, the cells were washed and put back into culture with fresh media. GFP percentage was checked by flow cytometry 3 days later. The GFP^+^ CD56-APC^+^ cells were sorted using BD FACS AriaFusion.

### Degranulation Assay

2 × 10^5^ NK-92 cells were co-incubated with 2 × 10^5^ target cells in a final volume of 200 μl in V-bottomed 96-well plate at 37°C and 5% CO_2_ for 4 h. In blocking experiments, target cells were pre-incubated with the corresponding 25 μg/mL blocking antibodies [anti-CD155 (clone SKII.4, Biolegend), anti-DNAM1 (clone DX11, BD Pharmingen) and anti-NKG2D (clone 1D11, Biolegend)] for 15 min on ice prior to co-culture. Fluorochrome-conjugated anti-CD107a-PE (H4A3, BD Biosciences) mAb was added at the initiation of the assay. After 1 h of co-incubation, GolgiStop (BD Biosciences) was added at a 1:300 dilution. The cells were then washed, resuspended in ice-cold PBS and stained with surface anti-CD56 (NCAM 16.2, APC, or BV421, BD Biosciences or Biolegend) to be analyzed by flow cytometry. For flow cytometry, cells were first gated on FSC-A vs. SSC-A, followed by gating on single cells via FSC-A vs. FSC-H, then GFP^+^CD56^+^ cells were selected and lastly CD107a^+^CD56^+^ percentage was recorded for analysis. All flow cytometry analysis was performed with FlowJo software v10.5 (BD Biosciences).

### Microscopy-Based Cellular Cytotoxicity

Image cytometry evaluation of NK cell cytotoxicity using calcein-acetoxymethylester (calcein-AM) fluorescent dyes has been previously reported to provide comparable sensitivity to traditional Cr^51^-release assays, while simultaneously providing morphological information and avoiding the use of radioactive materials (31, 32).

Tumor cells were seeded in flat-bottom 96-well CellBind plates (Corning) at a density of 1 × 10^4^-2 × 10^4^ cells/per well and cultured for 24–48 h. When the desired confluence was reached, the assay was initiated and cells were washed with HBSS and stained with 40 μl of 10 μM Calcein Red-AM (Thermo Fisher Scientific) and 1 μg/ml Hoescht-33342 (Thermo Fisher Scientific) for 30 min at 37°C. Cells were washed twice with HBSS before adding 50 μl fresh phenol red-free DMEM assay media. Probenecid (2 μM) was added to staining and assay medias to reduce dye efflux. NK-92 cells were additionally counted and resuspended in the same assay media. NK-92 cells (50 μl) were added to target cells at a 10:1 or 5:1 E:T ratio based on the number of seeded tumor cells and plates were incubated at 37°C for 4–6 h. Images were then collected using the CellInsight CX7 High-Content Imaging System (Thermo Fisher Scientific). Using a 10X objective, four fields of view were collected per well, with conditions run in triplicate. Images were analyzed using HCS Studio Software (Thermo Fisher Scientific). Briefly, tumor cells were identified as DAPI positive nuclei bounded by a Calcein Red positive cytoplasmic border. Apoptotic bodies were size-excluded from the analysis. Tumor nuclei were further distinguished based on their size and shape compared to NK-92 cell nuclei. Parameters were validated across all patient samples for all conditions and at 4 or 6 h timepoints. Tumor cell viability was assessed by determining the average fluorescent intensity (AFI) of individual tumor cells within each well. Percent viability was calculated by comparing the AFI of each condition to non-treated controls. Results are reported as the mean viability of two independent experiments. Results were analyzed using a one-way ANOVA with Tukey's *post-hoc* analysis.

### Analysis of NK Cells Cytotoxicity by Xcelligence RTCA

Real-time cell viability experiments were performed using the xCELLigence RTCA DP device (ACEA Biosciences) placed in a humidified incubator at 37°C and 5% CO_2_. The E-16 plates were incubated with 100 μL of cell-free growth medium [10% and 15% FBS containing McCoy's 5A medium (GE)] at room temperature for 15 min. After incubation, background impedance signal was measured to control all the connections. The target cells were seeded into plates at 5 × 10^3^ in 100 μL for U-2 OS and Saos-2 cell lines. The plates were mounted to the device after incubation at room temperature for 30 min before starting the experiment. The target cells were allowed to settle for 15–17 h before adding effector cells. The following day, the effector cells were added onto the target cells at an E:T ratio of 1:1. Real time measurements were performed by recording the Cell index (CI) every 15 min for a period of 40 h. Data analysis was carried out with the RTCA software (version 1.2, Roche Diagnostics).

### Statistical Analysis

For preparation of graphs and statistical analysis, GraphPad Prism v.7.0 (GraphPad Software Inc.) was used.

## Results

### Lymphocyte Characterization in Human Sarcomas and Matched Peripheral Blood Reveals a Defect in the Tumor-Infiltrating NK Cell Compartment

**Figure 1:**
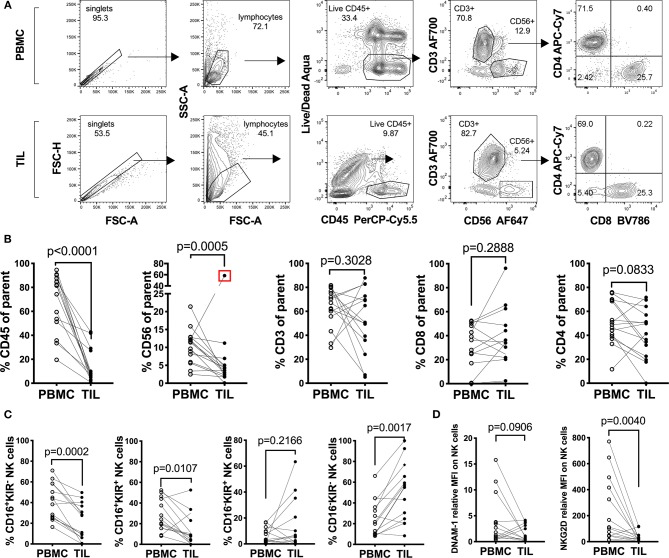
Characterization of peripheral and tumor-infiltrating T and NK cell populations from freshly isolated sarcoma patient material. **(A)** Representative gating strategy and **(B)** percentages of leukocytes: CD45^+^, T_helper/regulatory_ cells: CD3^+^CD4^+^, T_cytotoxic_ cells: CD3^+^CD8^+^ and NK cells: CD3^−^CD56^+^ among fresh PBMC and TIL of sarcoma patients (*n* = 14). The Live/Dead exclusion gate also includes markers CD14-V500 and CD19-V500 as a dump channel **(C)** Percentages of the Q1: CD16^+^KIR^−^, Q2: CD16^+^KIR^+^, Q3: CD16^−^KIR^+^, and Q4: CD16^−^KIR^−^ of NK cells from PBMC and TIL of sarcoma patients from **(C)** (*n* = 14). **(D)** Relative Median Fluorescence Intensity of DNAM-1 and NKG2D expression on NK cells in PBMC and TIL of sarcoma patients (*n* = 14). Statistical analysis was performed by Wilcoxon matched pairs signed rank non-parametric *t*-test and the Grubb's outlier test. In **(B,C)** the outlier is marked with a red square and the *p*-value is based on statistical analysis upon exclusion of the outlier.

In order to understand the immunological landscape of sarcomas and to assess possible immune defects that may contribute to the poor clinical responses observed, we characterized the major tumor-infiltrating leukocyte (TIL) populations and compared their frequencies to matched PBMCs from the same patients (Figure 1 and Table 1). A decrease in the overall percentage of TILs was observed in all patients compared to matched PBMCs (*n* = 14, Wilcoxon test, *p* = 0.0078) (Figure 1B). This was primarily reflected by a significant decrease in the CD3^−^CD56^+^ NK cell compartment (*p* = 0.0005), while no clear trend was observed between tumor-infiltrating and peripheral CD3^+^ bulk T cells, CD3^+^CD4^+^ and CD3^+^ CD8^+^ T cells (Figure 1B).

**Table 1:** List of sarcomas used in this study.

**Tumor code**	**Tumor type**	**Age**	**NK cell ligand profiling**		**PBMC vs. TILs info**	**NK cell screening**
			**Fresh**	**Propagated**		
HTT10	Synovial Sarcoma	40–45		+		+
HTT12	Extraoseos Osteosarcoma	85–90		+		+
HTT15	Rhabdomyosarcoma	5–10		+		+^*^
HTT16	Pleomorphic Myxofibrosarcoma	75–80		+		+
HTT17	Osteosarcoma	15–20		+		+
HTT21	Myxofibrosarcoma	35–40		+		+
HTT25	Pleaomorphic Spindle Cell Sarcoma	80–85		+		+
HTT26	Chondrosarcoma	70–75		+		+
HTT29	Chondrosarcoma	25–30		+		+
HTT30	Chondrosarcoma	55–60		+		+
HTT31	High Grade Pleomorphic Fibrosarcoma	70–75		+		+
HTT38	ST sarcoma	85–90		+		+
HTT39	Osteosarcoma	20–25		+		+
HTT41	Fibrosarcoma	40–45		+		
HTT42	Fibrosarcoma	70–75		+		
HTT45	Chondrosarcoma	75–80		+		
HTT46	Chindrosarcoma	75–80		+		
HTT47	Ewing's sarcoma	30–35		+		
HTT48	Pleomorphic Myxofibrosarcoma	75–80		+		
HTT49	Unknown subtype	65–70		+		
HTT50	Ewing's sarcoma	10–15	+	+	+	
HTT51	Myxofibrosarcoma	70–75	+			
HTT52	Myxofibrosarcoma	70–75	+	+	+	
HTT53	Myofibrosarcoma, High Grade	55–60	+	+		+^*^
HTT54	Myxoliposarcoma	50–55		+		
HTT55	Myxoid liposarcoma	70–75	+	+	+	+^*^
HTT57	Osteosarcoma	55–60	+	+	+	
HTT58	Leiomyosarcoma	60–65	+	+	+	
HTT61	Osteosarcoma	5–10		+	+	
HTT62	Chondrosarcoma	25–30		+	+	
HTT64	Synovial Sarcoma	30–35			+	
HTT67	Fibrosarcoma	0–5	+	+		
HTT71	Renal cell carcinoma	35–40			+	
HTT73	Ewing's sarcoma	75–80		+		+^*^
HTT77	Leiomyosarcoma	70–75			+	
HTT78	Osteosarcoma	5–10	+	+		
HTT79	Chondrosarcoma	65–70	+	+		+^*^
HTT80	Fibrosarcoma	80–85		+	+	
HTT81	Chondrosarcoma	55–60		+	+	
HTT82	Osteosarcoma	5–10	+	+	+	+^*^
HTT85	Chondrosarcoma	50–55			+	
HTT86	Pleomorphic fibroblastic sarcoma	50–55			+	

* *Only tested with WT and DNAM-1^+^ GM NK-92s*.

Since this pointed toward a general NK cell defect in sarcomas, we performed more detailed analysis of the surface receptor expression profile of the CD3^−^CD56^+^ population in tumors vs. PBMC. When assessing CD16 (FcγRIII) and collectively KIR2DL1 (CD158a), KIR2DL2/L3 (CD158b), and KIR3DL1(CD158e) in seven sarcoma patients, we observed a generalized decrease in the CD16^+^KIR^+^ and CD16^+^KIR^−^ NK cell population in the TILs compared to NK cells in matched PBMC (*n* = 14, *p* = 0.0002 and 0.0107, respectively) (Figure 1C; Figure S1A). A similar comparison was performed on the expression of activating receptors DNAM-1 (CD226) and NKG2D on CD3^−^CD56^+^ NK cells in TILs and PBMC of the same seven patients. DNAM-1 expression was decreased in 10 out of 14 TIL NK cells compared to PBMC, while the remaining four patients had increased expression of DNAM-1 on TIL NK cells (Figure 1D; Figure S1B). Lastly, 12 patients out of 14, exhibited decreased or unchanged expression of NKG2D on NK cells in the tumor compared to in PBMC (*p* = 0.040) (Figure 1D; Figure S1B). Overall, we observed minimal NK cell infiltration in sarcomas and the expression of activating receptors DNAM-1 and NKG2D was very low in both peripheral and TIL NK cells.

### NK Cell Ligand Expression Profiling of Fresh and *in vitro* Propagated Sarcoma Explants Identifies Common Expression of PCNA, CD112, and CD155

**Figure 2:**
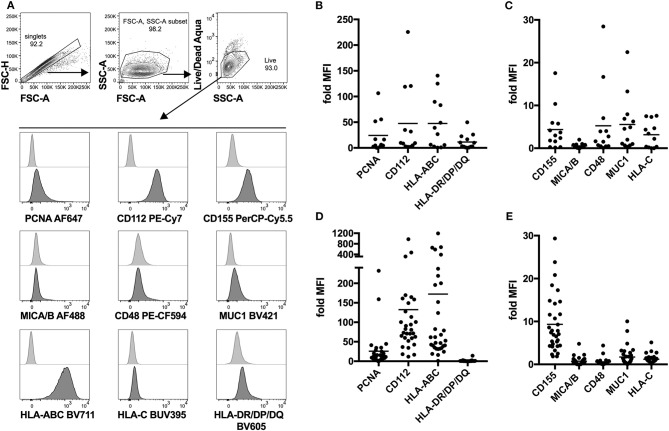
Lymphocyte ligand expression profile of fresh and *in vitro* propagated sarcoma explants. **(A)** Representative gating strategy for flow cytometry-based analysis of lymphocyte ligand surface expression on *in vitro* propagated sarcoma explants. In the case of fresh sarcoma explants, CD45-BV510 is included in the same channel as Live/Dead Aqua as an exclusion marker. **(B,C)** Fold MFI of lymphocyte ligands on fresh sarcoma explants. Normalization: (sample MFI – unstained MFI)/unstained MFI (*n* = 13). **(D,E)** Fold MFI of lymphocyte ligands on *in vitro* propagated sarcoma explants. Normalization: (sample MFI – unstained MFI)/unstained MFI (*n* = 32).

Next, we attempted to identify possible interaction partners between sarcoma tumor cells and NK cells which could account for the observed decrease in tumor-infiltrating NK cells. To do that, we performed detailed phenotyping of the lymphocyte ligands expressed on freshly isolated as well as *in vitro* propagated primary sarcoma explants, which we generated by serial passaging of cells from the freshly dissociated sarcomas (Table 1). Using a flow cytometry-based phenotyping approach, we assessed the expression of PCNA (NKp44 ligand), CD112 (DNAM-1 ligand), CD155 (DNAM-1 ligand), MICA/B (NKG2D ligand), CD48 (BLAST-1) (2B4 ligand), NTBA (NTBA ligand), MUC1 (Mucin 1) (Siglec-7 and−9 ligand), MHC Class I molecules HLA-ABC, and HLA-C (KIR2DL2/3 ligand), as well as MHC Class II molecules HLA-DR/DP/DQ (Figure 2A). Freshly isolated primary sarcomas expressed activating ligands CD112 and low levels of CD155 and CD48 as well as the inhibitory ligands PCNA, MUC1, HLA-ABC, and HLA-DR/DP/DQ (*n* = 13) (Figures 2B,C). Consistent with the analysis of the fresh sarcoma explants, the *in vitro* propagated primary sarcoma explants robustly expressed a similar but enhanced signature including the markers PCNA, CD112, CD155 to a lesser extent and HLA-ABC (*n* = 32) (Figures 2D,E). The phenotypes observed in sarcoma explants reveal that, despite the fact that NK cell infiltration in the tumor is limited and DNAM-1 expression on tumor infiltrating NK cells is low, CD112 and CD155 stand out as tangible targets for immunotherapies (Figures 1A,D).

### GM NK-92 Cells Overexpressing DNAM-1 or NKG2D Efficiently Degranulate Against Primary Sarcoma Explants

**Figure 3:**
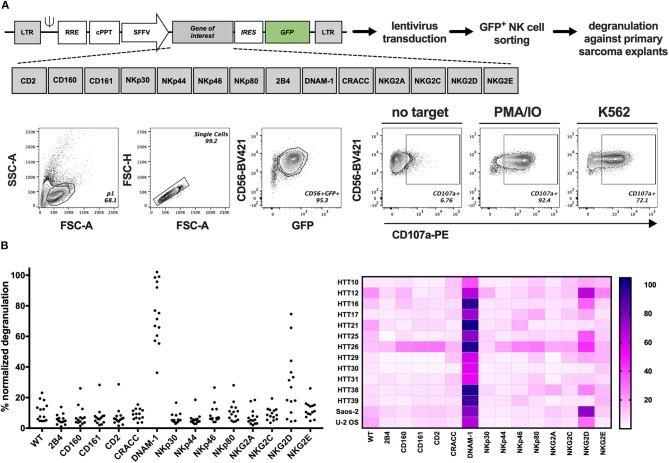
GM NK-92 cell-based screening platform tested against 12 different primary sarcoma explants. WT or GM NK-92 cells were co-cultured with target cells at 1:1 (E:T) ratio for 4 h. %CD56^+^CD107a^+^ NK-92 cells were analyzed by flow cytometry. PMA and ionomycin (PMA/IO) were used as positive stimulators of degranulation and K562 as the validated target of NK-92 cells. **(A)** Generation of GM NK-92 cells and gating strategy for all degranulation flow cytometry analysis. **(B)** Dot plots and heatmap showing normalized %CD56^+^CD107a^+^ WT or GM NK-92 cells against each sarcoma explant and sarcoma cell lines Saos-2 and U-2 OS (PMA/IO responses for each GM NK-92 cell line was set as 100% for the normalization of the data; results from one representative experiment, plotted as means of technical replicates).

The presence of several ligands for activating NK cell receptors prompted the analysis of functional NK cell responses against sarcoma cells. Therefore, we developed a screening platform that assesses the capacity of different NK cell activating receptors to efficiently trigger degranulation against target cells without prior knowledge of which ligands are present on the target cell surface. Genetically modified (GM) NK-92 cells over-expressing one NK receptor at a time were generated by introducing the gene-of-interest-IRES-GFP lentiviral constructs to NK-92 cells and sorting GFP^+^ cells containing the receptors depicted in Figure 3A and Figure S3. Sorted cells were expanded and used in a functional, cell-based screening approach to test which of them could be optimal candidates to target each primary sarcoma explant. GM NK-92 cells were co-cultured at 1:1 (E:T) ratio with 12 selected primary sarcoma explants (Table 1) and two well-established sarcoma cell lines (Saos-2 and U-2 OS) side by side with control NK-92 cells for comparative analysis of degranulation (Figure 3B; Figure S4). In line with the observed expression of DNAM-1 receptor ligands CD112 and CD155 (Figure 2C), DNAM-1^+^ GM NK-92 cells degranulated significantly against all 12 sarcoma explants and cell lines, while NKG2D^+^ GM NK-92 cells degranulated more than 20% to 10 out of 12 (*p*-values in Table S1). We further confirmed the triggering of cytokine production by DNAM-1^+^ and NKG2D^+^ NK-92 cells (Figure S6) upon culture with primary sarcoma explants (HTT12, HTT25, HTT31). Compared to WT NK-92 cells, DNAM-1^+^ and NKG2D^+^ NK-92 cells displayed enhanced production of TNFα and IFNγ upon co-culture with Saos-2 cells indicating that the GM NK cells have the capacity to trigger a wide range of effector functions. We also observed strong involvement of DNAM-1-mediated anti-tumor responses with 6 independent *in vitro* propagated sarcoma explants (HTT15, HTT53, HTT55, HTT73, HTT79, and HTT82) and 5 healthy donor PBMCs (Figure S5). As expected, tumors expressing CD112 and/or CD155 triggered a high degranulation response from only DNAM-1^+^ NK-92 cells while these only low/moderately responded in the case of healthy donor PBMCs. Thus, it can be concluded that overexpression of DNAM-1 on NK-92 cells directly increased degranulation and cytokine prodcution against *in vitro* propagated primary sarcoma explants bearing CD112 and/or CD155, while healthy PBMCs were minimally affected.

### DNAM-1 and NKG2D Are Responsible for Degranulation Against Primary Sarcoma Explants

To verify the direct contribution of the respective activating NK cell receptors in mediating the observed degranulation, we used blocking antibodies to the receptors on the NK-92 cells or the corresponding ligand on the target cells (CD155 in the case of DNAM-1), in order to interfere with their specific interaction.

**Figure 4:**
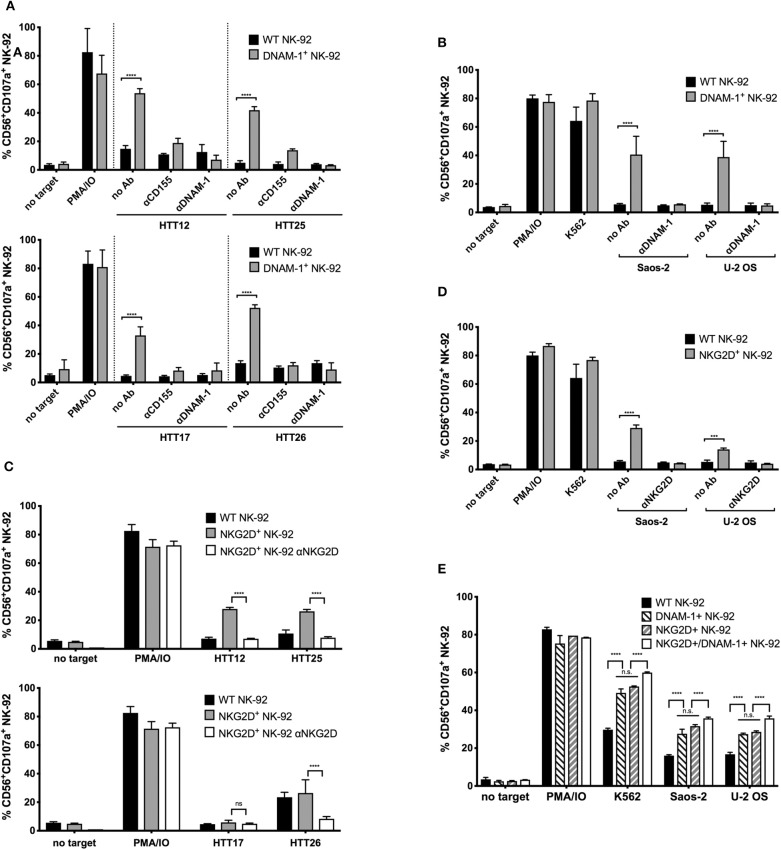
Enhanced degranulation of NKG2D^+^ and DNAM-1^+^ GM NK-92 cells against selected sarcoma explants and Saos-2 and U-2 OS cell lines is receptor mediated. CD155, DNAM-1, or NKG2D blocking was done 15 min prior to degranulation set up. The assay was set as previously described, at E:T ratio 1:1. **(A)** Anti-CD155 on tumors and anti-DNAM-1 blocking of WT or DNAM-1^+^ GM NK-92 cells against primary sarcoma explants and **(B)** anti-DNAM-1 blocking of WT or DNAM-1^+^ GM NK-92 cells against Saos-2 and U-2 OS cell lines. **(C)** Anti-NKG2D blocking of NKG2D^+^ GM NK-92 cells against primary sarcoma explants and **(D)** anti-NKG2D blocking of WT or NKG2D^+^ GM NK-92 cells against Saos-2 and U-2 OS cell lines (results from two independent experiments, means plotted with error bars indicating SD). **(E)** Comparison of degranulation by DNAM-1 or NKG2D GM NK-92 cells with NK-92 cells co-expressing both receptors against Saos-2 and U-2 OS cells (^****^ indicates *p* < 0.0001 with 2-way ANOVA analysis).

When DNAM-1^+^ GM NK-92 cells were treated with a blocking antibody against DNAM-1, degranulation responses against HTT12, HTT17, HTT25, and HTT26 were completely abolished (Figure 4A). When CD155 was blocked on the sarcoma explants, a residual response could still be observed by the DNAM-1^+^ GM NK-92 cells which is possibly due to the presence of the other ligand, CD112, on the surface of tumors. However, when DNAM-1 was blocked, all degranulation was abrogated. Hence, it was confirmed that the degranulation of GM NK-92 cells was only mediated through the engagement of DNAM-1 with either of its ligands on the sarcoma explants.

Similarly, degranulation of NK-92 cells overexpressing NKG2D was assessed against HTT12, HTT17, HTT25, and HTT26, in blocking experiments where the effector cells were incubated with a blocking antibody against NKG2D prior to co-incubation (Figure 4C). As expected, blocking of NKG2D abrogated the response to levels similar to that of no target controls for samples HTT12, HTT25, and HTT26. As expected, HTT17 which was not a target of NKG2D^+^ GM NK-92 cells, was not affected by the blocking.

Likewise, the blocking of DNAM-1 and NKG2D on NK-92 cells prior to co-incubation with targets, caused the respective responses of DNAM-1^+^ and NKG2D^+^ GM NK-92 cells to decrease to background levels when tested against Saos-2 and U-2 OS sarcoma cell lines (Figures 4B,D). Thus, it can be concluded that the degranulation responses of GM NK-92s over-expressing DNAM-1 and NKG2D are solely due to the abundant and functional interaction between the respective receptors on the NK-92 cells and the corresponding ligands on the sarcoma cells.

Having observed a relevant role for both DNAM-1 and NKG2D, we further analyzed whether the co-expression of the two receptors would synergize in triggering activity against sarcoma cells. For this purpose, we carried out a second genetic modification on NKG2D^+^ GM NK-92 cells using the DNAM-1 expression vector and used FACS sorting to enrich the NKG2D^+^/DNAM-1^+^ GM NK-92 cells. Post-sorting analysis of DNAM-1 and NKG2D single or co-expressing cells confirmed similar levels of receptor expression and assured that any observed functional differences would not be merely due to this (Figure S7). Analysis of degranulation against U-2 OS and Saos-2 cell lines by co-expressing NK-92 cells revealed a slight increase in degranulation activity (Figure 4E) in comparison to DNAM-1 or NKG2D single positive cells. Due to lack of synergistic effects with co-expression of both DNAM-1 and NKG2D, we did not proceed further with tests against primary sarcoma explants.

### DNAM-1^+^ GM NK-92 Cells Demonstrate Enhanced Cytotoxic Activity Against Primary Sarcoma Explants

**Figure 5:**
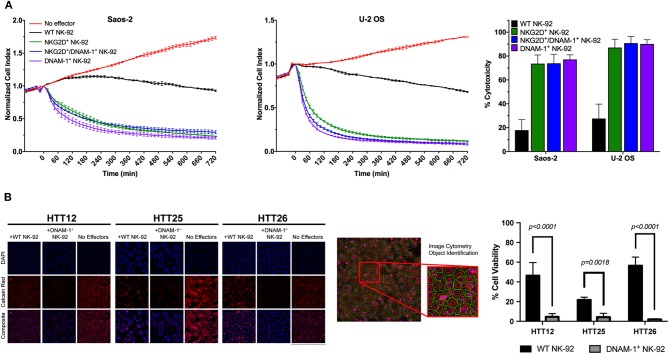
Evaluation of NK cell cytotoxicity. **(A)** Representative results from cytotoxicity assays on Saos-2 (left panel) and U-2 OS (middle panel) cell lines using the xCelligence RTCA platform. Calculation of cytotoxic activity after 4 h of co-culture from two independent experiments (right panel) shows enhanced cytotoxic activity by GM NK cells. **(B)** Live tumor cells were loaded with Calcein-AM dye and co-cultured with effector cells at a 10:1 E/T ratio and incubated for 4 h. Live tumor cells were identified as Hoescht positive nuclei bounded by a Calcein Red positive cytoplasmic border. Apoptotic bodies were size excluded from the analysis. Tumor cell viability was assessed by determining the average fluorescent intensity (AFI) of individual tumor cells within each well. Percent viability was calculated by comparing the AFI of each condition to non-treated controls. Results are reported as the mean viability of two independent experiments, with three technical replicates per experiment and four fields of view per well. Results were analyzed using a two-way ANOVA with Tukey's *post-hoc* analysis in GraphPad Prism. DNAM-1^+^ NK-92 cells were found to significantly reduce the viability of primary sarcoma patient tumor cells, *p* < 0.0001 (HTT12, HTT26); *p* = 0.0018 (HTT25).

While NK cell degranulation is a prerequisite for direct NK cell-mediated cytotoxicity, it does not necessarily correlate with target cell lysis. In order to observe the functional consequences of the slightly increased levels of degranulation in NK-92 cells co-expressing DNAM-1 and NKG2D, we performed electrical impedance-based cytotoxicity assays against U-2 OS and Saos-2 cells (Figure 5A). Quantification of cytotoxic activity at 4 h revealed that DNAM-1 single positive NK-92 cells are the most efficient in killing U-2 OS and Saos-2 cells. While both other NK-92 cell lines also rapidly killed target cells at a much higher rate compared WT NK-92 cells, the co-expression of the two receptors did not significantly enhance cytotoxic activity (Figure 5A). Rather, we observed that co-expressing NK-92 cells do not show as high cytotoxicity as DNAM-1 single positive NK-92 cells. Therefore, we conclude that the co-expression of DNAM-1 and NKG2D does not seem to be a feasible approach in further enhancing the anti-sarcoma activity of NK cells.

Furthermore, we used a calcein-AM image cytometry assay to visually validate target cell killing by WT or GM NK-92 cells. While testing of NKG2D^+^ GM NK-92 cells against sarcoma explants did not yield any significant results (Figure S8), DNAM-1^+^ GM NK-92 cells exhibited significantly increased cytotoxicity compared to WT-NK-92 cells against all three sarcoma explants (HTT12, HTT25, and HTT26), which were simultaneously tested in the same assay (Figure 5A). Representative images from three primary sarcoma explants co-cultured with WT and DNAM-1^+^ GM NK-92 cells for 4 h (E:T ratio 10:1) show that target cells in the spontaneous control (without effector cells) exhibited brightly fluorescent live cells. While there is some variability between samples, fewer fluorescent cells with diminished intensity were observed when co-cultured with WT-NK-92 cells. Overall, DNAM-1^+^ GM NK-92 cells exerted increased cytotoxic activity compared to WT controls in the imaging-based assays, supporting the degranulation results obtained and putting forward DNAM-1 as a prominent candidate in sarcoma immunotherapy.

### DNAM-1 and NKG2D Ligand Expression Is Associated With Poor Survival in Sarcoma

In order to investigate the individual roles of NKG2D ligands in the response of GM NK-92 cells, we primarily determined mRNA level expression of ULBP1, ULBP2, ULBP3, ULBP4, and ULBP5 molecules in the sarcoma explants used in Figure 3 and complemented this data with MICA/B cell surface expression and NKG2D-Fc staining of each explant (Figure S2). Overall, we observe that the tumors differentially express various NKG2D ligands as was also confirmed with NKG2D-Fc staining. Interestingly, mRNA expression did not always correlate with cell surface NKG2D-Fc staining which may be due to a deficiency in membrane trafficking or shedding of ULBP molecules (33). Given the current data, it is difficult to speculate which one of the NKG2D ligands is the most potent engager of anti-tumor responses.

**Figure 6:**
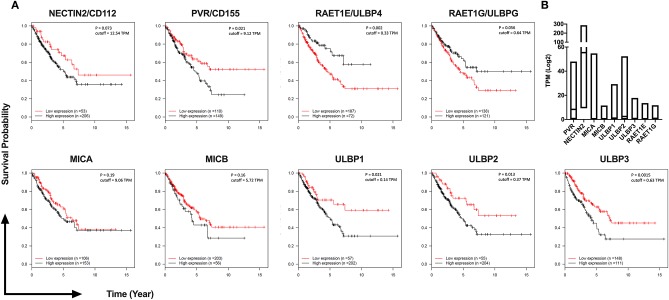
TCGA analysis of 259 Sarcoma patients reveals high expression of DNAM-1 and NKG2D ligands are associated with poor survival. **(A)** Kaplan-Meier survival estimation analysis results from 259 TCGA Sarcoma TPM values based on expression of DNAM-1 ligands (Nectin 2 or CD112, PVR or CD155) and NKG2D ligands (MICA, MICB, ULPB1, ULPB2, ULBP3, RAET1E, or ULBP4 and RAET1G or ULBP5). Data from 20th to 80th percentile was compared. **(B)** Log2 TPM value of ligands of NKG2D and DNAM-1 from TCGA Sarcoma expression data.

To investigate the potential clinical significance of individual NKG2D and DNAM-1 ligands, we analyzed their expression levels in 259 sarcoma samples available in The Cancer Genome Atlas (TCGA) database of the National Cancer Institute (Figure 6). We observed that the high expression of DNAM-1 ligand CD155 as well as the high expression of NKG2D ligands ULBP1, ULBP2, and ULBP3 are negatively associated with survival in sarcoma patients. Taken together with our observation of decreased DNAM-1 and NKG2D expression on tumor-infiltrating NK cells in sarcoma and the enhanced cytotoxic activity of NK-92 cells expressing DNAM-1 or NKG2D against sarcoma explants, these results put forward DNAM-1- or NKG2D-based immunotherapy as a potentially effective approach in sarcoma treatment.

### DNAM-1^+^ and NKG2D^+^ GM NK-92 Cells Provide a Novel Approach for Efficiently Targeting a Wide Range of Solid and Hematological Malignancies

**Figure 7:**
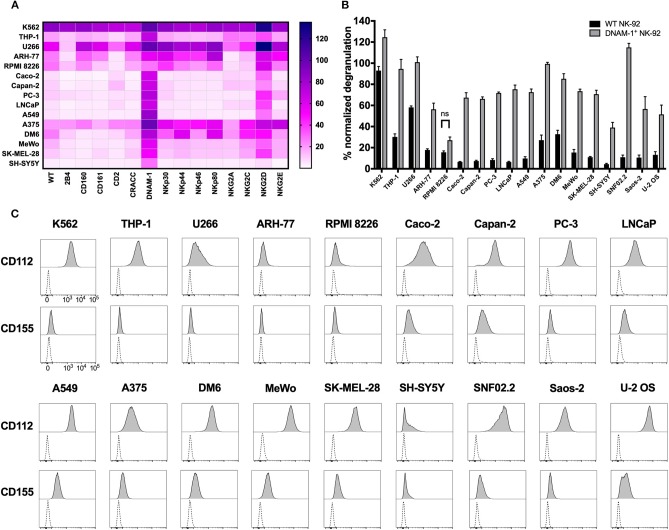
Cancer cell lines expressing CD112 and/or CD155 are targeted by DNAM-1^+^ NK-92 cells. **(A)** Degranulation response of all GM NK-92 cells and **(B)** of WT or DNAM-1^+^ NK-92 cells in 4 h at 1:1 (E:T) with indicated cell lines were normalized according to the PMA/ionomycin response of effectors in each run [**A**: a single representative, **B**: Means of two independent experiments, run in duplicates are depicted with error bars indicating SEM. All except for RPMI 8226 response give significance (*p* < 0.0001 with 2-way ANOVA analysis.)] **(C)** Cancer cell lines were stained for surface expression of CD112 and CD155 and analyzed by flow cytometry (dashed line: unstained, filled histogram: stained). ns, not significant.

Identification of the role of DNAM-1 and NKG2D in boosting NK cell responses against sarcoma using functional screening with GM NK-92 cells (Figures 3, 4; Figure S4), encouraged us to further evaluate the use of this approach against other solid and hematological malignancies (Figure 7A; Table 2). We assessed the degranulation capacity of all 14 GM NK-92 cell lines against various well-established cancer cell lines, including the metastatic prostate carcinomas PC-3 and LNCaP, primary pancreatic ductal adenocarcinoma Capan-2, primary colorectal adenocarcinoma Caco-2, primary lung alveolar basal epithelial adenocarcinoma A549, metastatic neuroblastoma SH-Sy5y, metastatic nerve sheath tumor SNF02.2, melanomas SK-MEL-28, MeWo, A375 and DM6, myelomas U266, ARH-77 and RPMI 8226 and leukemias K562 (CML), and THP-1 (AMoL) (Figure 7A; Table 2). Assessment of degranulation responses with the GM NK-92 cell-based screening platform showed that NKG2D^+^ GM NK-92 cells had enhanced degranulation against majority of the cell lines except for SH-SY5Y (Figure 7A). In line with this observation, previous studies and the human protein atlas database demonstrated moderate/high expression of at least one NKG2D ligand in all cell lines used except in the neuroblastoma cell line SH-SY5Y (35–41). Importantly, identification of differential receptor-mediated responses against tested cells lines such as DM6, THP-1, and ARH-77 cell lines, validates the functionality of each receptor introduced to NK-92 cells, and further demonstrates the applicability of the NK cell-based screening platform for the identification of tumor type- and most importantly patient-specific targetable NK cell receptor/ligand interactions (Figure 7A).

**Table 2:** List of cell lines used in this study.

**Cell line**	**Origin**	CD112 (fold MFI^*^)	CD155 (fold MFI^*^)	**WT NK-92 (%CD56^**+**^CD107a^**+**^)**	**DNAM-1^**+**^NK-92 (%CD56^**+**^CD107a^**+**^)**
K562 (ATCC^®^ CCL-243™)	Chronic myelogenous leukemia	99.05	1.36	71.7 (±5.9)	81.3 (±6.5)
THP-1 (ATCC^®^ TIB-202™)	Acute monocytic leukemia	64.83	0.94	23.4 (±6.7)	60.7 (±11.7)
U266 (ATCC^®^ TIB-196™)	Myeloma; plasmacytoma	10.82	2.14	38.65 (±5)	69.8 (±9.3)
RPMI8226 (ATCC^®^ CCL-155™)	Myeloma; plasmacytoma	1.16	1.24	10.5 (±2.9)	18.5 (±3.6)
ARH-77 (ATCC^®^ CRL-1621™)	Plasma cell leukemia	3.22	0.59	11.7 (±0.4)	39.1 (±9.4)
Caco-2 (ATCC^®^ HTB-37™)	Primary colorectal adenocarcinoma	59.73	4.06	4.7 (±1.1)	46 (±10.3)
Capan-2 (ATCC^®^ HTB-80™)	Primary pancreatic ductal adenocarcinoma	50.92	4.73	5.3 (±1.9)	45.4 (±9.4)
PC-3 (ATCC^®^ CRL-1435™)	Metastatic prostate adenocarcinoma	60.7	1.44	6.8 (±2.8)	52.9 (±0.7)
LNCaP (ATCC^®^ CRL-1740™)	Metastatic prostate carcinoma	18.60	2.25	4.6 (±1.2)	47.5 (±7.3)
A549 (ATCC^®^ CCL-185™)	Primary lung alveolar basal cell epithelial adenocarcinoma	85.31	6.69	7.7 (±3.6)	41.5 (±7.9)
A375 (ATCC^®^ CRL-1619™)	Malignant melanoma	18.28	2.71	17.6 (±5.8)	59.9 (±4)
DM6 (34)	Malignant melanoma	51.67	2.76	23.3 (±7.5)	53.5 (±7)
SK-MEL-28 (ATCC^®^ HTB-72™)	Malignant melanoma	60.45	4.75	7.9 (±2.1)	36.8 (±8.3)
MeWo (ATCC^®^ HTB-65™)	Malignant melanoma	120.91	5.58	11.4 (±5.8)	47.4 (±16.9)
SH-SY5Y (ATCC^®^ CRL-2266™)	Metastatic neuroblastoma	7.89	3.64	3.2 (±0.7)	21.7 (±3.1)
sNF02.2 (ATCC^®^ CRL-2885™)	Metastatic malignant peripheral nerve sheath tumor (MPNST)	106.87	4.40	11 (±4)	115 (±7.7)
Saos-2 (ATCC^®^ HTB-85™)	Osteosarcoma	59.46	5.28	9.4 (±4.9)	43.8 (±16.4)
U-2 OS (ATCC^®^ HTB-96™)	Osteosarcoma	425.32	15.59	11.8 (±6.1)	39.9 (±12.2)

* *Calculated as (MFI_stained_ – MFI_unstained_)/MFI_unstained_*.

Moreover, DNAM-1^+^ GM NK-92 cells also showed overall enhanced responses compared to WT control except against RPMI 8226, while the range of the response varied greatly depending on the different target cell types (Figure 7A). Further validation of the superior degranulation capacity of DNAM-1^+^ GM NK-92 cells compared to WT controls, was independently assessed for all other types of cancers and showed that all cell lines induced significantly enhanced degranulation responses upon engagement of the DNAM-1^+^ GM NK-92 cells, except for RPMI 8226 (Figure 7B).

Since CD112 and/or CD155 expression profiles of some of the cell lines used in this study have not been fully identified previously, we next assessed the surface expression of CD112 and CD155 by flow cytometry (Figure 7C; Table 2). The general increase observed in anti-tumor responses exerted by DNAM-1^+^ GM NK-92 cells was in line with surface expression of at least one of the ligands on the surface of the cancer cell lines. With the exception of target cell line RPMI 8226, where surface expression of CD112 and CD155 were very low, all degranulation responses by DNAM-1^+^ GM NK-92 cells were significantly enhanced compared to WT NK-92 (*p* < 0.0001).

Overall, our results suggest that arming NK-92 cells with activating receptors such as DNAM-1 and NKG2D can boost anti-tumor responses against various different malignancies. Likewise, identification of the unique NK cell receptor response profile of various tumors highlights the potential importance of the cell-based screening in the identification of targetable patient-specific NK cell/tumor interactions.

## Discussion

In this study, our primary aim was to identify potential NK cell/sarcoma interactions that can be modulated or targeted to enhance NK cell anti-tumor responses. To achieve this, we have assessed the immune profile of freshly isolated sarcoma explants, TILs and matched PBMCs, and developed a GM NK-92 cell line-based screening platform to identify the potential functional effects of different NK cell receptors in anti-sarcoma responses. Using this platform for the first time, we have characterized tumor-specific functional NK cell receptor signatures of primary human sarcomas as well as well-established cancer cell lines. Briefly, we have observed that NK cells minimally infiltrated sarcomas and expression of DNAM-1 and NKG2D were very low in both peripheral and sarcoma-infiltrating NK cells (Figure 1), even though ligand expression was observed in freshly dissociated tumors (Figure 2) and the expression profiles of DNAM-1 and NKG2D ligands were associated with poor survival in sarcoma patients (Figure 6). On the other hand, GM NK-92 cells that overexpress DNAM-1 or NKG2D efficiently targeted sarcoma explants (Figure 3) and various established tumor cell lines (Figure 7), while WT NK-92 cells failed to respond in general. The response of GM NK-92 cells was strictly dependent on the corresponding receptor and ligands (Figure 4) and cytotoxicity was further confirmed with imaging-based cytotoxicity assays (Figure 5).

Our results provide an essential insight into how to enhance NK cell mediated anti-sarcoma responses, while confirming for the first time the feasibility of a GM NK-92 cell-based functional screening approach in developing and predicting personalized immunotherapies for cancer patients. Given the clinical applicability of NK-92 cells (42, 43), it is also possible to generate off-the shelf GM therapeutic NK-92 libraries and predict the most efficient treatment option for each individual using such an *in vitro* functional screen carried out on tumor biopsies. This rapidly personalized adoptive immunotherapy regimen can then be used as monotherapy as well as in combination with other already available approaches, such as checkpoint inhibition.

Compelling evidence supports an important role for the immune system in the disease pathogenesis as well as in anti-tumor responses against sarcomas. This also suggests that immunotherapies would be a promising treatment alternative for sarcoma patients, but surprisingly they have been inadequately explored. Clinical trials using T cell receptor (TCR) modified T cells seem to focus on NY-ESO-I on subtypes of sarcomas, such as liposarcoma and synovial sarcoma (NCT03450122, NCT01477021, NCT01343043, NCT03399448) (16, 44) while applications of Chimeric Antigen Receptor (CAR) technology in sarcoma include HER2 (NCT00902044), EGFR (NCT03618381), CD44v6 and GD2 (45) directed CARs. However, downregulation of tumor-associated antigens is a common mechanism that tumors use to escape immune recognition, which has been shown to occur in both soft tissue and bone sarcomas (46–48), highlighting the potential importance of NK cell-based immunotherapies in the cases where antigen-specific responses fail (49, 50).

While NK cells are one of the promising candidates in the development of advanced cancer immunotherapies (51–54), very few clinical trials are currently exploring NK cells as a therapeutic option for sarcomas and none are exclusively designed to be sarcoma-specific (NCT02100891, NCT01875601). Nevertheless, sarcomas comprise over 100 different subtypes (55) and this diversity has made it very difficult to predict or customize efficient immunotherapies. This study provides a novel perspective for the development of efficient sarcoma-specific immunotherapies through the identification of DNAM-1 and NKG2D as well as their respective ligands as potential therapeutic targets for various subtypes of sarcomas and also enhancing NK cell mediated anti-sarcoma responses by DNAM-1^+^ and NKG2D^+^ GM NK cells.

Additionally, as a new angle to introduce antigen-specific recognition to NK cells, we and others recently published a novel approach to arm an NK cell line with a TCR in order to target tumors with high specificity while at the same time overcoming the problem of endogenous TCR mispairing (56, 57). Combining expression of antigen-specific receptors and tumor-specific NK cell receptors to further enhance anti-tumor responses by dual arming stands out as one of the future reflections of this study. NK cells that can be armed to enhance adaptive and innate anti-tumor responses are also inherently equipped to detect loss of MHC-I expression and get activated through disengagement of KIR/MHC-I mediated inhibitory signaling, thus potentially providing a back-up plan in case tumors downregulate MHC-I due to the TCR-mediated immune pressure. The persistence and multi-targeting potential of genetically enhanced NK cells can be further fine-tuned by inhibition of the tumor and the suppressive tumor microenvironment using, for example, checkpoint blockade therapy and inducing antigen-specific anti-tumor responses using antigen-specific monoclonal antibodies that induce CD16-mediated NK cell activation and antibody-dependent cellular cytotoxicity (ADCC).

DNAM-1 and NKG2D are two activating NK cell receptors which recognize stress-induced ligands that are commonly over-expressed by tumors. Thus, they have been implicated as key players in immunity against human tumors and have been extensively explored in multiple approaches to cancer immunotherapy (58). Many studies have shown reduced expression of activating receptors like DNAM-1 and NKG2D on TILs from cancer patients (59, 60) or shedding of their respective ligands from the tumor cells (61, 62). In line with this, we observed very low expression of DNAM-1 and NKG2D both on peripheral and on tumor-infiltrating NK cells (Figure 1D). The downregulation of NK cell activating receptors in the periphery may result in diminished anti-tumor responses and can be reversed by *ex vivo* activation of NK cells expressing DNAM-1 and NKG2D, that have been shown to efficiently target and kill Ewing sarcoma (EWS), rhabdomyosarcoma (RMS) and osteosarcoma cell lines (derived from patient tumor samples) *in vitro* (27, 63, 64). On the other hand, here we demonstrate that primary human sarcomas (Figure 3) and other tumors (Figure 7) can be efficiently targeted by genetically modified NK-92 cells overexpressing NKG2D or DNAM-1 receptors.

Moreover, while previous studies have demonstrated expression and clinical relevance of CD112 and CD155 mostly from a sarcoma subset-oriented perspective (24, 63, 65), here we simultaneously assessed the expression of CD112 and CD155 in a diverse group of primary human sarcomas. The restricted expression of these molecules in healthy tissues, combined with the expression on all sarcoma explants provides valuable information regarding potential new targets for the development of targeted immunotherapies for sarcoma (Figure 2). DNAM-1^+^ GM NK-92 cells degranulated against all primary sarcoma explants as well as against the majority of the established cell lines, proving the potential of CD112 and CD155 as tumor-specific markers for targeted immunotherapies (Figures 3, 7). This, along with the absence of the degranulation response of NK cells against healthy donor PBMCs, showed that the use of DNAM-1^+^ GM NK-92 cells could be restricted/directed only to targets that had elevated CD112 and/or CD155 expression (Figures 2, 7), which is observed in tumors from various origins (65–83), including osteosarcomas (24).

As previously mentioned, NKG2D expression was very low on TIL NK cells but also on PBMC of sarcoma patients (Figure 1D), as was the expression of NKG2D ligands MICA/B on both fresh and propagated sarcoma explants (Figures 2C,E). This could be a result of peripheral immunosuppression induced by the tumor through NKG2D ligand shedding as has been observed in colon adenocarcinoma patients (84). While NKG2D is expressed on both T and NK cells, few attempts have been made to sensitize T cells to tumors through the NKG2D signaling axis (85, 86). However, NKG2D plays a significant role in tumor cell immune recognition and in particular in the perforin-mediated cytolytic response of NK cells (87). Moreover, NKG2D ligands are abundantly overexpressed in several human malignancies (88–93) and sensitize tumors to NK cell-mediated killing (94–99). Thus, it comes as no surprise that tumors employ a wide array of mechanisms to modulate NKG2D ligand expression to escape NK cell immune surveillance (100–106). Additional approaches to the one we describe here, have focused on developing therapeutics that increase the expression of NKG2D on NK cells (27, 107, 108) or enhance the expression levels of NKG2D ligands, as has been tested for Ewing sarcoma cell lines (109, 110).

Similar to the DNAM-1^+^ GM NK-92 cells, using NKG2D^+^ GM NK-92 cells, we were able to identify a subset of sarcoma explants that also induced efficient degranulation albeit to a lower degree (Figure 3B). NKG2D^+^ GM NK-92 cells also showed efficient degranulation responses against several of the well-established tumor cell lines of various types of malignancies (Figure 7). Degranulation was NKG2D-specific as the use of blocking antibodies to the receptor abrogated the responses (Figures 4C,D). This could be explained by the presence of other NKG2D ligands such as the ULBP family of proteins which are also expressed by the U-2 OS and Saos-2 sarcoma cell lines (40). The approach described here, identifies NKG2D as an additional receptor that can arm NK cells to successfully target patient tumor cells expressing the relevant ligands.

We also addressed whether the co-expression of DNAM-1 and NKG2D would further enhance the anti-sarcoma activity of NK-92 cells. NKG2D and DNAM-1 are known to use similar activation motifs and downstream signaling of the receptors overlap significantly, sharing involvement of essential molecules to exert their effector functions (111–113). Thus, it is possible to argue that the co-triggering of DNAM-1 and NKG2D may result in the enhancement of these signals but also has the risk of the two signals stumbling upon a bottleneck of either ligand engagement or signaling intermediates that dictates the amount of activation possible. Our results indicate that while the co-expression of the two receptors seems to slightly affect degranulation, it did not provide a significant enhancement in cytotoxic activity (Figures 4E, 5A). A third scenario including synergistic effects would theoretically be more possible if the second receptor made use of distinct signaling pathways, as in the case of 2B4, TRAIL, and FASL, but such an approach remains to be analyzed in further studies.

Overall, while tumor-infiltrating NK cells have decreased expression of DNAM-1 and NKG2D, sarcoma tumors highly express DNAM-1 and NKG2D ligands. Thus, we propose that genetically modified NK cells overexpressing DNAM-1 and/or NKG2D can be used to target these tumors efficiently and overcome the observed NK cell deficiency in sarcomas. Moreover, we have also demonstrated that the higher expression of many DNAM-1 or NKG2D ligands significantly associate with poor survival of sarcoma patients (Figure 6). Taking this information into account, it is possible to suggest that treatment with DNAM-1 and/or NKG2D expressing NK cells would especially benefit patients with bad prognosis.

In an era when personalized medicine is continuously gaining momentum, the use of biomarkers to identify the most efficient course of treatment is becoming increasingly necessary. To that end, efforts to develop new approaches that enable quick and efficient generation of important information regarding a patient's individual tumor phenotype have intensified in recent years. The functional screening platform described in this study enables the identification of NK cell receptor reactivities against a variety of different types of sarcomas, as well as many other cancer cell lines. Consequently, the NK-92 cell-based screening platform could function as a tool to perform parallel assessment of several activating NK cell receptors and identify the ones with the ability to arm NK-92 cells for cytotoxic activity against the individual patients' tumor cells. This would provide important information that could contribute to making immunotherapy treatments patient-customized and more efficient.

While the *in vivo* anti-tumor activity and treatment efficiency of DNAM-1^+^ or NKG2D^+^ GM NK-92 cells yet remains to be investigated in xenograft models of sarcoma, we and others have demonstrated that WT and GM NK-92 cells have the ability to exert potent anti-tumor responses both in pre-clinical animal models and clinical trials including various solid tumors (114–118) as well as several hematological malignancies (119–122). Both, modified and unmodified NK-92 cells have shown successful treatment responses and are currently under investigation for numerous indications. Quick and robust expansion of the relevant GM NK-92 cells to large numbers facilitates their use as a standardized off-the-shelf therapeutic that is safe, efficient and highly specific to the patient's tumor. Ultimately, the described approach can provide prognostic value through the identification of potent tumor/NK cell interactions, but also GM NK-92 cells that overexpress DNAM-1 or NKG2D can also be used in the clinical setting to treat cancer patients.

## Data Availability Statement

The datasets generated for this study are available upon request to the corresponding author.

## Author Contributions

MC, EA, TS, and AD contributed to the conception and design of the NK screening platform. TS designed lentiviral constructs based on LeGO vectors. MC and RK produced virus and did transductions. KK sorted genetically modified cells. ES, MC, DO, and BJ maintained cell culture of all NK and target cell lines. HT provided deidentified surgical material. AD, A-MG, and HT designed the clinic-to-bench pipeline. ES, RK, MH, AD, and A-MG did tumor dissociation and PBMC isolations. ES, RK, and MH maintained sarcoma serial passaging and stocking. A-MG, AD, and TC ran flow cytometry of sarcoma (fresh/propagated) and patient blood samples. MC, ES, and DO ran degranulation assays with GM NK-92 cells. DO and CP has generated GM NK cell lines, sorted, and performed Xcelligence assays. RP performed qPCR. MA, AM, and CZ performed analysis of TCGA data. BJ and A-MG ran imaging-based cellular cytotoxicity assays. A-MG performed co-culture experiments for intracellular cytokine detection in NK-92 cells upon tumor co-culture. ES and A-MG analyzed data, prepared figures, and wrote the first draft of the manuscript. BJ, DO, and AD analyzed data and prepared figures. ES, A-MG, AD, TS, BJ, and MC edited and finalized the manuscript. All other authors contributed to the final editing of the manuscript.

### Conflict of Interest

The authors declare that the research was conducted in the absence of any commercial or financial relationships that could be construed as a potential conflict of interest.
