# Supplementary material for: Boosting Natural Killer Cell-Mediated Targeting of Sarcoma Through DNAM-1 and NKG2D
Source: Front Immunol. 2020 Jan 28;11:40. doi: 10.3389/fimmu.2020.00040 (PMC7001093; doi:10.3389/fimmu.2020.00040)
Supplement: Supplementary file 1 [file Data_Sheet_1.PDF]

Supplementary Materials

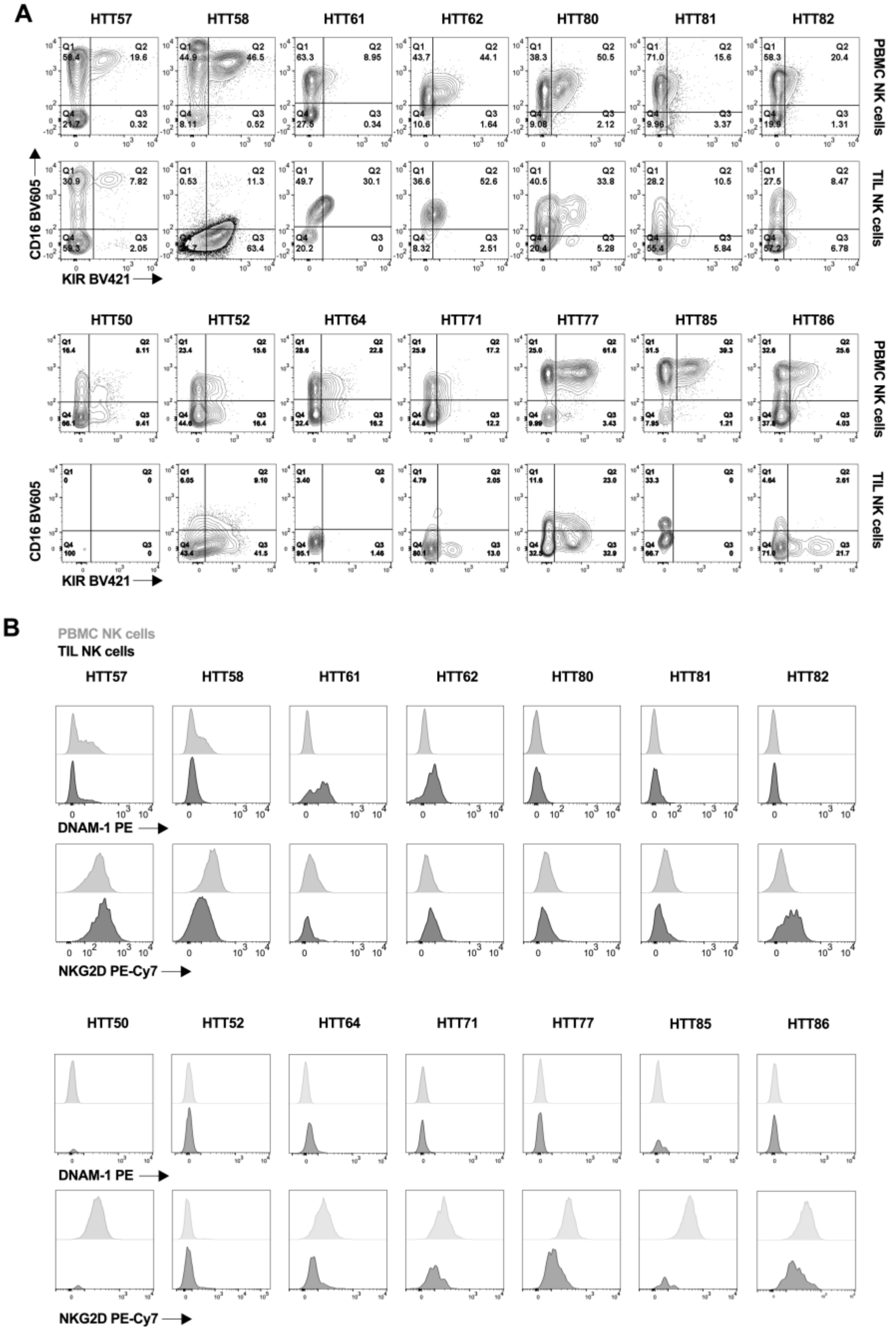

Figure S1. Characterization of receptor expression profile on peripheral and tumor-infiltrating NK cells from freshly isolated sarcoma patient material. (A) Contour plots showing expression of CD16 and KIRs (CD158a; KIR2DL1, CD158b; KIR2DL2/L3 and CD158e1; KIR3DL1) on CD56^{+} NK cells in PBMC versus NK cells in TILs of sarcoma patients assessed by flow cytometry (n=14). **(B)**

Histograms showing median fluorescence intensity of DNAM-1 and NKG2D on NK cells in PBMC versus TILs of sarcoma patients assessed by flow cytometry (n=14).

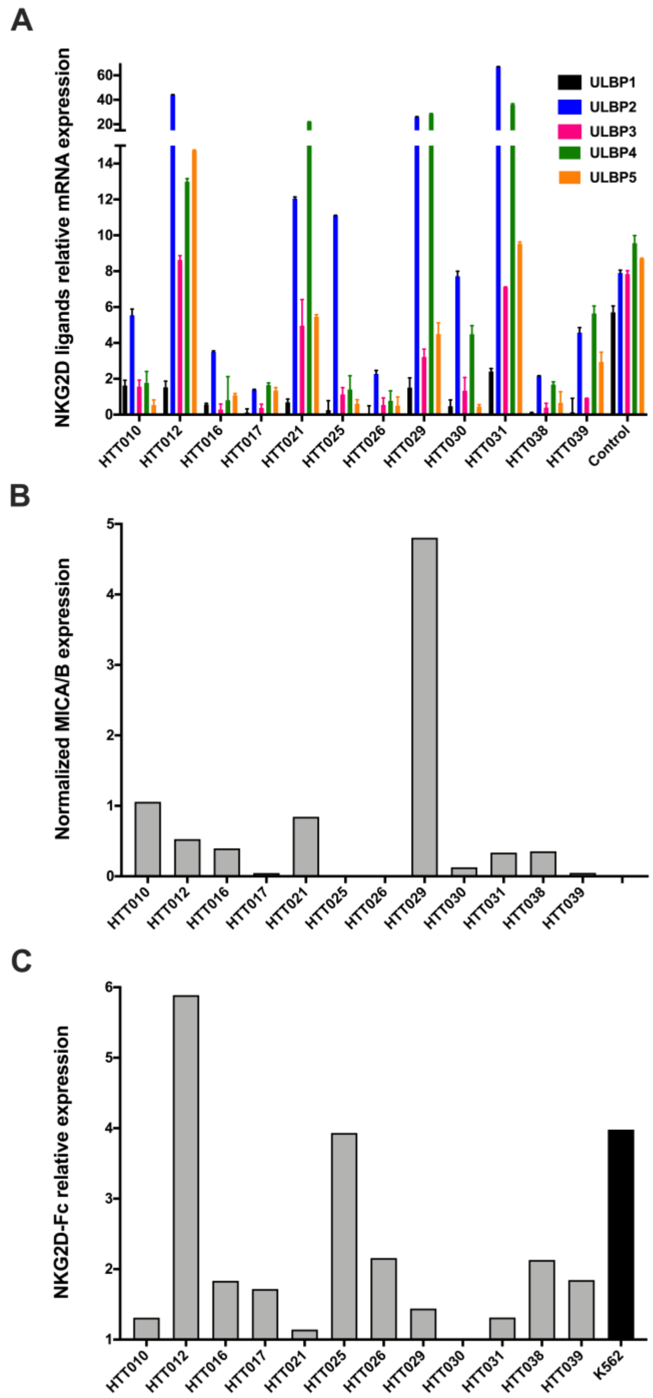

Figure S2. Characterization of NKG2D ligand expression of sarcomas on by qPCR and flow cytometry. (A) qPCR data regarding ULBP molecule expression and **(B)** surface expression of MICA/B assessed by flow cytometry and **(C)** surface staining for flow cytometry-based detection

of binding to NKG2D ligands using NKG2D-Fc chimeric protein. Data shows relative fold expression.

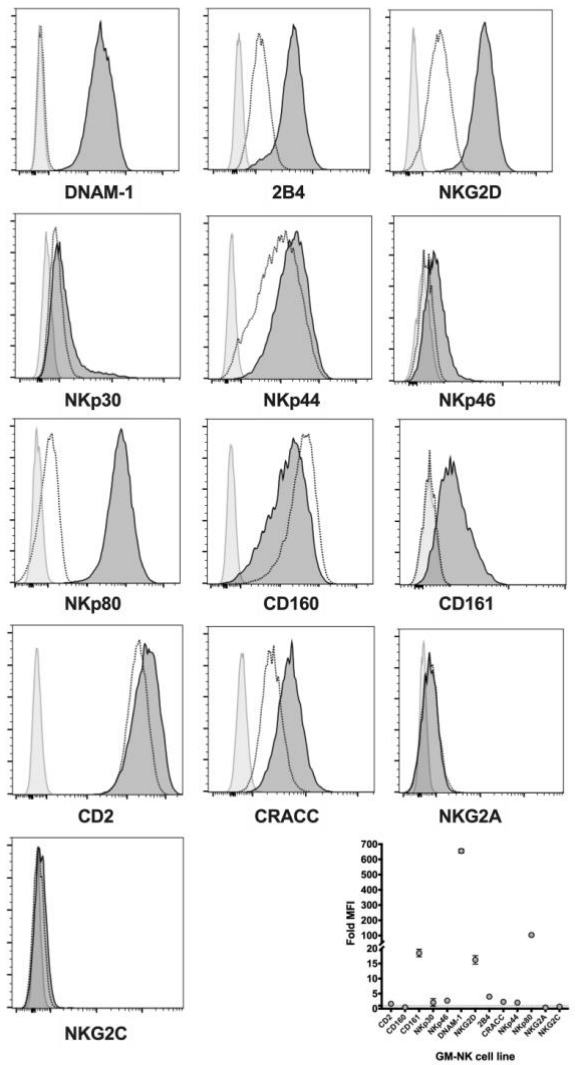

Figure S3. GM NK-92 surface expression of corresponding receptors as validated by flow cytometry. WT or GM NK-92 cells were stained with relevant surface receptors and mean fluorescence intensity of each receptor staining were obtained by flow cytometry. Fold MFI was calculated by [GM NK-92 ($MFI_{stained}$-$MFI_{unstained}$)/($MFI_{unstained}$)]/ [WT NK-92 ($MFI_{stained}$-$MFI_{unstained}$)/($MFI_{unstained}$)]. Error bar indicates SD of two separate stainings.

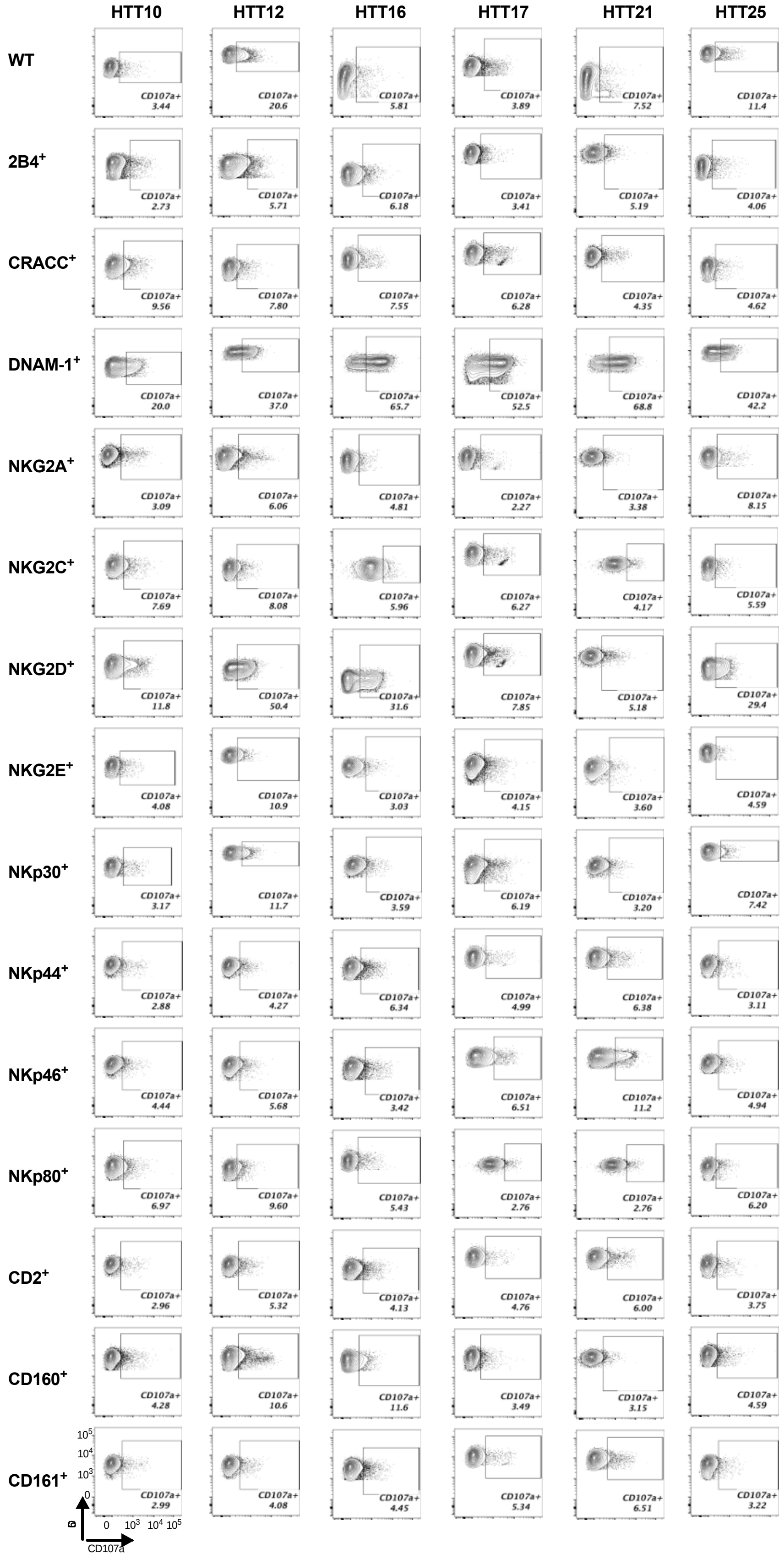
HTT10
HTT12
HTT16
HTT17
HTT21
HTT25
WT: CD107a+ 3.44; CD107a+ 20.6; CD107a+ 5.81; CD107a+ 3.89; CD107a+ 7.52; CD107a+ 11.4
2B4+: CD107a+ 2.73; CD107a+ 5.71; CD107a+ 6.18; CD107a+ 3.41; CD107a+ 5.19; CD107a+ 4.06
CRACC+: CD107a+ 9.56; CD107a+ 7.80; CD107a+ 7.55; CD107a+ 6.28; CD107a+ 4.35; CD107a+ 4.62
DNAM-1+: CD107a+ 20.0; CD107a+ 37.0; CD107a+ 65.7; CD107a+ 52.5; CD107a+ 68.8; CD107a+ 42.2
NKG2A+: CD107a+ 3.09; CD107a+ 6.06; CD107a+ 4.81; CD107a+ 2.27; CD107a+ 3.38; CD107a+ 8.15
NKG2C+: CD107a+ 7.69; CD107a+ 8.08; CD107a+ 5.96; CD107a+ 6.27; CD107a+ 4.17; CD107a+ 5.59
NKG2D+: CD107a+ 11.8; CD107a+ 50.4; CD107a+ 31.6; CD107a+ 7.85; CD107a+ 5.18; CD107a+ 29.4
NKG2E+: CD107a+ 4.08; CD107a+ 10.9; CD107a+ 3.03; CD107a+ 4.15; CD107a+ 3.60; CD107a+ 4.59
NKp30+: CD107a+ 3.17; CD107a+ 11.7; CD107a+ 3.59; CD107a+ 6.19; CD107a+ 3.20; CD107a+ 7.42
NKp44+: CD107a+ 2.88; CD107a+ 4.27; CD107a+ 6.34; CD107a+ 4.99; CD107a+ 6.38; CD107a+ 3.11
NKp46+: CD107a+ 4.44; CD107a+ 5.68; CD107a+ 3.42; CD107a+ 6.51; CD107a+ 11.2; CD107a+ 4.94
NKp80+: CD107a+ 6.97; CD107a+ 9.60; CD107a+ 5.43; CD107a+ 2.76; CD107a+ 2.76; CD107a+ 6.20
CD2+: CD107a+ 2.96; CD107a+ 5.32; CD107a+ 4.13; CD107a+ 4.76; CD107a+ 6.00; CD107a+ 3.75
CD160+: CD107a+ 4.28; CD107a+ 10.6; CD107a+ 11.6; CD107a+ 3.49; CD107a+ 3.15; CD107a+ 4.59
CD161+: CD107a+ 2.99; CD107a+ 4.08; CD107a+ 4.45; CD107a+ 5.34; CD107a+ 6.51; CD107a+ 3.22
10^5
10^4
10^3
0
0 10^3 10^4 10^5
CD107a

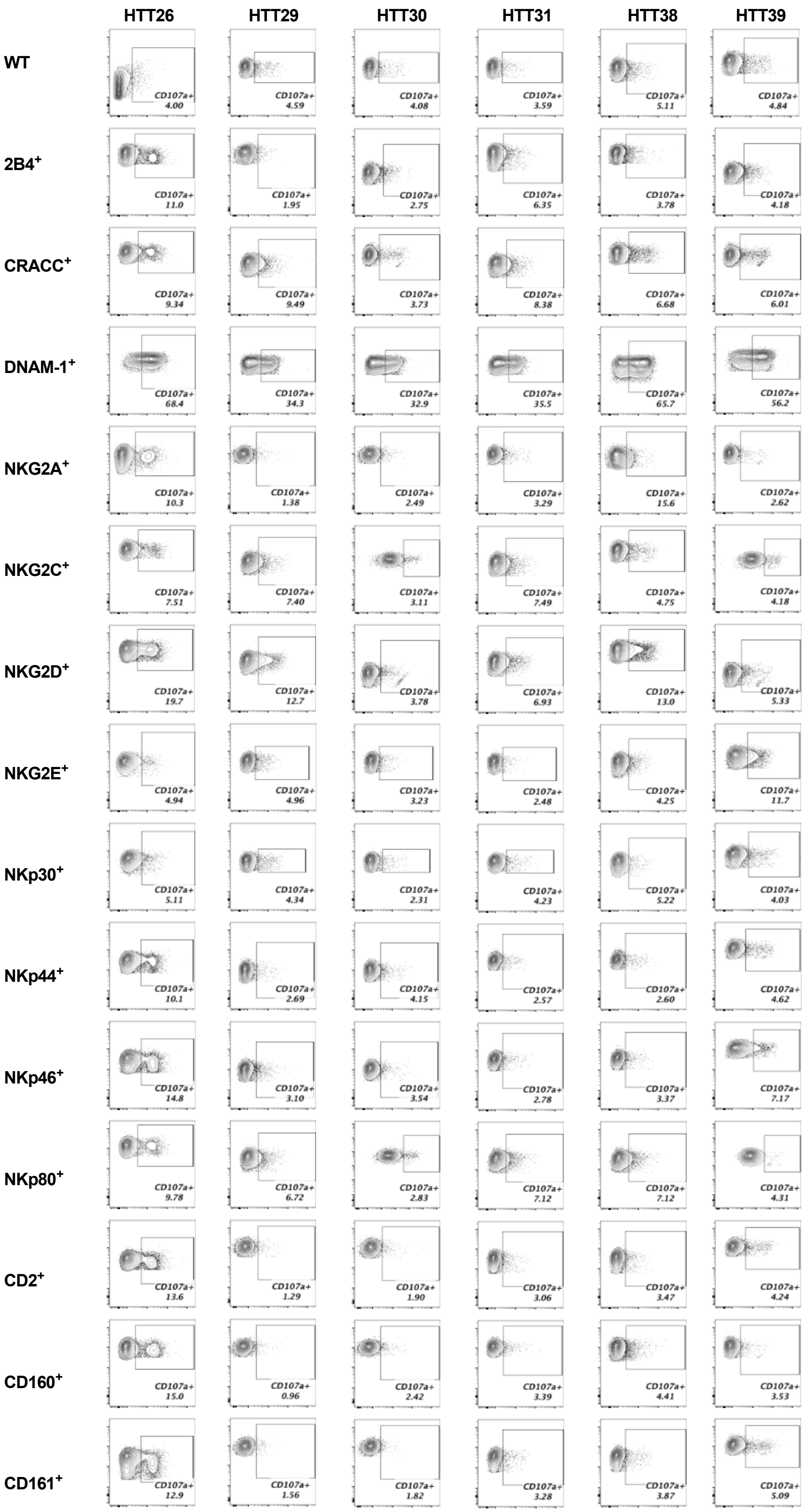

HTT26
HTT29
HTT30
HTT31
HTT38
HTT39
WT
CD107a+ 4.00
CD107a+ 4.59
CD107a+ 4.08
CD107a+ 3.59
CD107a+ 5.11
CD107a+ 4.84
2B4+
CD107a+ 11.0
CD107a+ 1.95
CD107a+ 2.75
CD107a+ 6.35
CD107a+ 3.78
CD107a+ 4.18
CRACC+
CD107a+ 9.34
CD107a+ 9.49
CD107a+ 3.73
CD107a+ 8.38
CD107a+ 6.68
CD107a+ 6.01
DNAM-1+
CD107a+ 68.4
CD107a+ 34.3
CD107a+ 32.9
CD107a+ 35.5
CD107a+ 65.7
CD107a+ 56.2
NKG2A+
CD107a+ 10.3
CD107a+ 1.38
CD107a+ 2.49
CD107a+ 3.29
CD107a+ 15.6
CD107a+ 2.62
NKG2C+
CD107a+ 7.51
CD107a+ 7.40
CD107a+ 3.11
CD107a+ 7.49
CD107a+ 4.75
CD107a+ 4.18
NKG2D+
CD107a+ 19.7
CD107a+ 12.7
CD107a+ 3.78
CD107a+ 6.93
CD107a+ 13.0
CD107a+ 5.33
NKG2E+
CD107a+ 4.94
CD107a+ 4.96
CD107a+ 3.23
CD107a+ 2.48
CD107a+ 4.25
CD107a+ 11.7
NKp30+
CD107a+ 5.11
CD107a+ 4.34
CD107a+ 2.31
CD107a+ 4.23
CD107a+ 5.22
CD107a+ 4.03
NKp44+
CD107a+ 10.1
CD107a+ 2.69
CD107a+ 4.15
CD107a+ 2.57
CD107a+ 2.60
CD107a+ 4.62
NKp46+
CD107a+ 14.8
CD107a+ 3.10
CD107a+ 3.54
CD107a+ 2.78
CD107a+ 3.37
CD107a+ 7.17
NKp80+
CD107a+ 9.78
CD107a+ 6.72
CD107a+ 2.83
CD107a+ 7.12
CD107a+ 7.12
CD107a+ 4.31
CD2+
CD107a+ 13.6
CD107a+ 1.29
CD107a+ 1.90
CD107a+ 3.06
CD107a+ 3.47
CD107a+ 4.24
CD160+
CD107a+ 15.0
CD107a+ 0.96
CD107a+ 2.42
CD107a+ 3.39
CD107a+ 4.41
CD107a+ 3.53
CD161+
CD107a+ 12.9
CD107a+ 1.56
CD107a+ 1.82
CD107a+ 3.28
CD107a+ 3.87
CD107a+ 5.09

Figure S4. Representative contour plots from each degranulation flow cytometry experiment.

Based on the gating strategy explained in Fig.3, here are the flow cytometry plots showing %$CD56^+CD107a^+$ NK-92 cells.

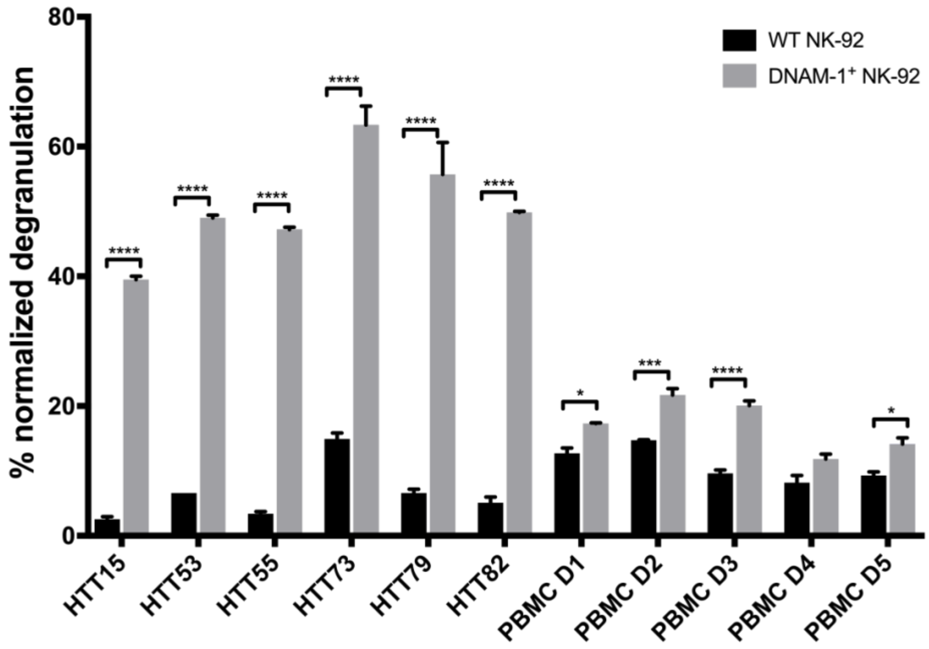

Figure S5. Degranulation of WT and DNAM-1$^+$ NK-92 cells against 6 other sarcoma explants and 5 healthy donor PBMCs. WT or DNAM-1$^+$ NK-92 cells were co-cultured with sarcoma explants or PBMCs at 1:1 (E:T) ratio for 4 hours. %CD56$^+$CD107a$^+$ NK-92 cells were analyzed by flow cytometry. PMA and ionomycin (PMA/IO) were used as positive stimulators of degranulation and K562 as the validated target of NK-92 cells. (PMA/IO responses for each GM NK-92 cell line was set as 100% for the normalization of the data; results from one representative experiment, plotted as means of technical replicates).

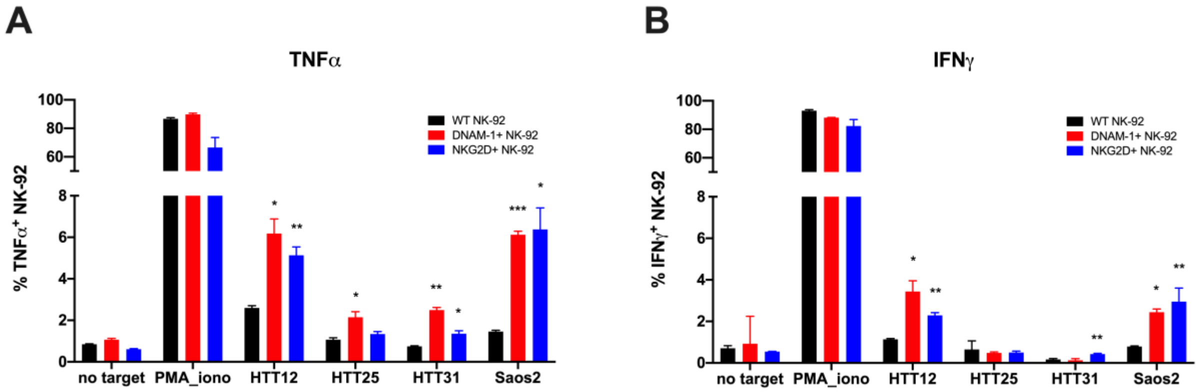

Figure S6. Sarcoma cells trigger cytokine production on DNAM-1$^+$ and NKG2D$^+$ GM NK-92 cells. **(A)** Percentage of TNF-α^+ and **(B)** IFN-γ^+ cells as assessed by flow cytometry after co-culture with indicated primary sarcoma explants and Saos-2 cell line. Representative results from two independent experiments, means plotted with error bars indicating SD. (* $p<0.05$; ** $p<0.01$; *** $p<0.001$; *** $p<0.0001$, 2-way ANOVA with Dunnet test)

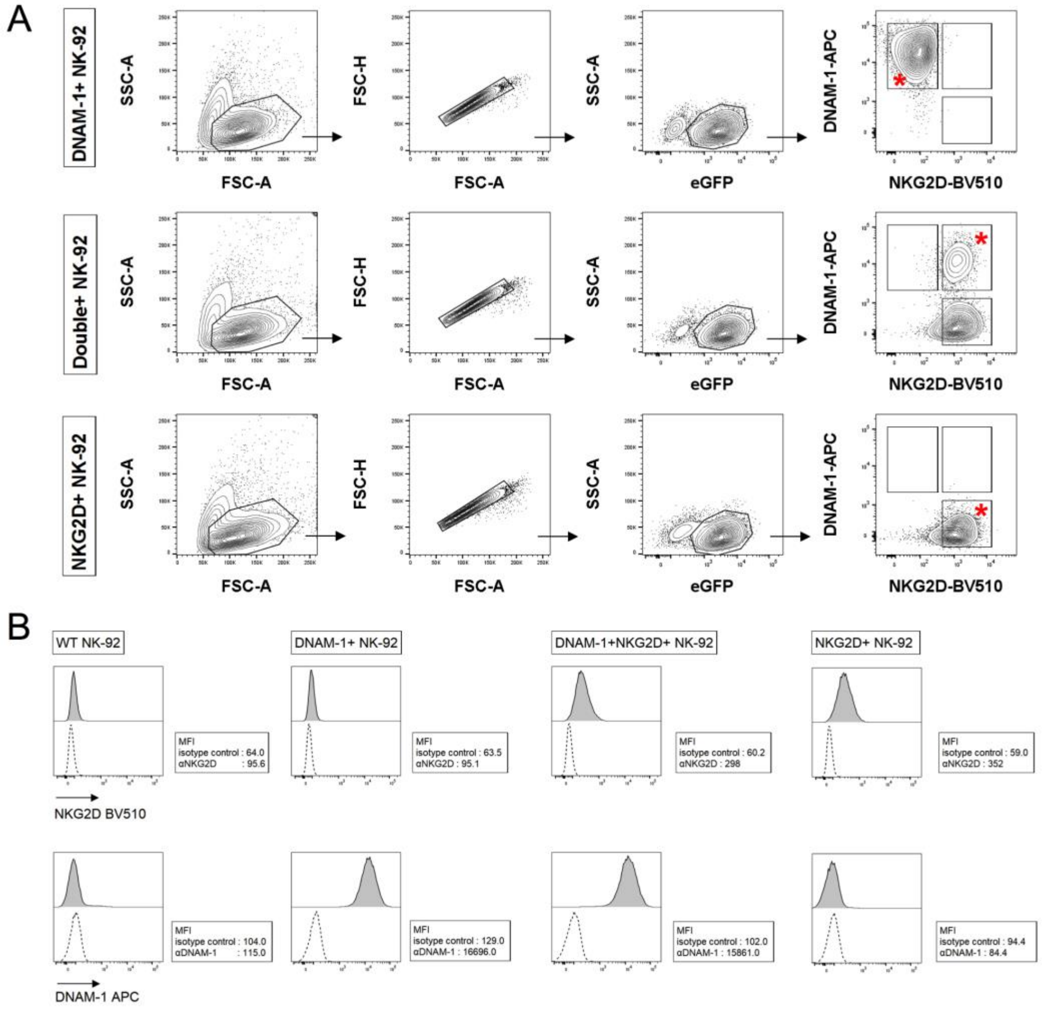

Figure S7: Sorting/post sorting of DNAM-1⁺ and NKG2D⁺ GM NK-92 cells. Genetic modification of NK-92 cells for NKG2D, DNAM-1 and co-expression. (A) Genetically modified NK-92 cells were sorted 7 days post lentiviral transduction. NKG2D⁺ and/or DNAM-1⁺ cell populations (shown by red asterisk) were sorted with fluorescence-activated cell sorting (FACS) by gating on FSC-A vs SSC-A, followed by single cells via FSC-A vs FSC-H, then eGFP vs SSC-A and lastly, on NKG2D vs DNAM-1 using BD FACS Aria II. (B) WT and GM NK-92 cells were stained with isotype control or αCD56, αNKG2D and αDNAM-1 antibodies and MFI of each receptor staining was calculated on flow cytometry (dashed line: isotype control, filled histogram: stained).

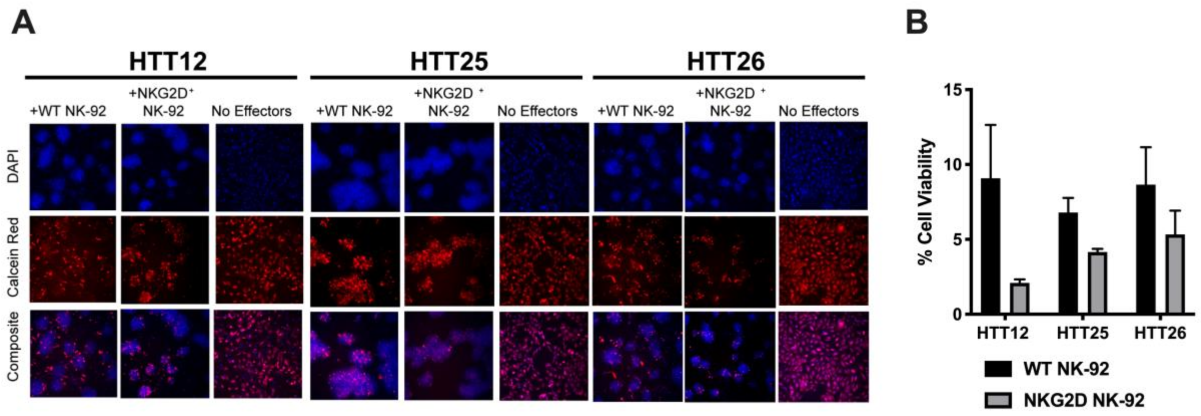

Figure S8: Image cytotoxicity of NKG2D NK-92 cells. Live tumor cells were loaded with Calcein-AM dye and co-cultured with effector cells at a 5:1 E/T ratio and incubated for 6 hours. Live tumor cells were identified as Hoescht positive nuclei bounded by a Calcein Red positive cytoplasmic border. Apoptotic bodies were size excluded from the analysis. Tumor cell viability was assessed by determining the average fluorescent intensity (AFI) of individual tumor cells within each well. Percent viability was calculated by comparing the AFI of each condition to non-treated controls. Results are reported as the mean viability of 3 technical replicates 4 fields of view per well. Results were analyzed using a two-way ANOVA with Tukey's posthoc analysis in GraphPad Prism.

Tumor code	Tumor type	Sex	Age	Degranulation p-value WT vs DNAM-1	Degranulation p-value WT vs NKG2D
HTT10	Synovial Sarcoma	M	43	**** p 0.0001	**** p 0.0001
HTT12	Extraoseos Osteosarcoma	M	89	**** p 0.0001	**** p 0.0001
HTT16	Pleomorphic Myxofibrosarcoma	M	79	**** p 0.0001	**** p 0.0001
HTT17	Osteosarcoma	M	15	**** p 0.0001	**** p 0.0001
HTT21	Myxofibrosarcoma	M	36	**** p 0.0001	**** p 0.0001
HTT25	Pleaomorphic Spindle Cell Sarcoma	M	83	**** p 0.0001	**** p 0.0001
HTT26	Chondrosarcoma	F	71	**** p 0.0001	**** p 0.0001
HTT29	Chondrosarcoma	M	25	**** p 0.0001	**** p 0.0001
HTT30	Chondrosarcoma	M	58	**** p 0.0001	n.s.
HTT31	High Grade Pleomorphic Fibrosarcoma	M	73	**** p 0.0001	* p 0.0395
HTT38	ST sarcoma	M	89	**** p 0.0001	**** p 0.0001
HTT39	osteosarcoma	F	22	**** p 0.0001	n.s.

Supplementary Table I. Degranulation p-values for WT vs DNAM-1 and WT vs NKG2D GM NK-92 cells in normalized degranulation graph given in Fig.3, analyzed by 2-way ANOVA.

Antibody target	Conjugation	Clone	Vendor	Assay
CD155	PerCP-Cy5.5	SKII.4	Biolegend	Flow Cytometry
MICA/MICB	AF488	6D4	Biolegend	Flow Cytometry
PCNA	AF647	PC10	Biolegend	Flow Cytometry
CD112	PE/CY7	TX31	Biolegend	Flow Cytometry
CD45	PerCP-Cy5.5	HI30	Biolegend	Flow Cytometry
NKG2D	PE/CY7	1D11	Biolegend	Flow Cytometry
NKG2D	BV510	1D11	Biolegend	Flow Cytometry
CD158e1 (KIR3DL1)	BV421	DX9	Biolegend	Flow Cytometry
CD16	BV605	3G8	Biolegend	Flow Cytometry
CD56	AF647	5.1H11	Biolegend	Flow Cytometry
2B4	PerCP Cy5.5	C1.7	Biolegend	Flow Cytometry
NKp46	BV605	9E2	Biolegend	Flow Cytometry
NKp80	APC	5D12	Biolegend	Flow Cytometry
CRACC	PE	162.1	Biolegend	Flow Cytometry
CD2	APC	RPA-2.10	Biolegend	Flow Cytometry
CD160	AF647	BY55	Biolegend	Flow Cytometry
CD161	BV605	HP-3G10	Biolegend	Flow Cytometry
CD48	PE-CF594	TU145	BD Biosciences	Flow Cytometry
MUC1 (CD227)	BV421	HMFG2	BD Biosciences	Flow Cytometry
HLA-DR, DP, DQ	BV605	Tu39	BD Biosciences	Flow Cytometry
HLA-ABC	BV711	G46-2.6	BD Biosciences	Flow Cytometry
HLA-C	BUV395	DT-9	BD Biosciences	Flow Cytometry
CD4	BB515	SK3	BD Biosciences	Flow Cytometry
CD158a (KIR2DL1)	BV421	HP-3E4	BD Biosciences	Flow Cytometry
CD158b (KIR2DL2/L3)	BV421	CH-L	BD Biosciences	Flow Cytometry
CD14	V500	MΦP9	BD Biosciences	Flow Cytometry
CD19	V500	HIB19	BD Biosciences	Flow Cytometry
CD3	AF700	UCHT1	BD Biosciences	Flow Cytometry
DNAM-1	PE	DX11	BD Biosciences	Flow Cytometry
DNAM-1	APC	11A8	Biolegend	Flow Cytometry
CD107a	PE	H4A3	BD Biosciences	Degranulation
CD56	APC	NCAM16.2	BD Biosciences	Degranulation/FC
CD56	BV421	NCAM16.2	BD Biosciences	Degranulation/FC
CD56	BV510	NCAM16.2	BD Biosciences	Degranulation/FC
CD56	BV421	1D11	Biolegend	Flow Cytometry
NKp30	BV421	P30-15	BD Biosciences	Flow Cytometry
NKp44	AF647	P44-8	BD Biosciences	Flow Cytometry
NKG2A	AF647	131411	R&D Systems	Flow Cytometry
NKG2C	APC	134591	R&D Systems	Flow Cytometry

Supplementary Table II. List of antibodies used in flow cytometry experiments.
